# Transcriptomic Analysis of Biofilm Formation Inhibition by PDIA Iminosugar in *Staphylococcus aureus*

**DOI:** 10.3390/antibiotics14070668

**Published:** 2025-07-01

**Authors:** Anna Tomusiak-Plebanek, Łucja Kozień, Estelle Gallienne, Maciej Florczyk, Sławomir Ciesielski, Piotr Heczko, Magdalena Strus

**Affiliations:** 1 Chair of Microbiology, Department of Bacteriology and Parasitology, Jagiellonian University Medical College, 31-121 Krakow, Poland; a.tomusiak@uj.edu.pl (A.T.-P.); lucja.kozien@gmail.com (Ł.K.); piotr.heczko@uj.edu.pl (P.H.); 2 Institut de Chimie Organique et Analytique (ICOA), UMR 7311, Université d’Orléans & CNRS, 45067 Orleans, France; estelle.gallienne-boivineau@univ-orleans.fr; 3 Laboratory of Molecular Neurobiology, Nencki Institute of Experimental Biology of the Polish Academy of Sciences, 02-093 Warsaw, Poland; m.florczyk@nencki.edu.pl; 4 Department of Environmental Biotechnology, University of Warmia and Mazury in Olsztyn, 10-907 Olsztyn, Poland; slawomir.ciesielski@uwm.edu.pl

**Keywords:** biofilm, iminosugar PDIA, *Staphylococcus aureus*, transcriptomic analysis

## Abstract

**Background:** Iminosugars are natural or synthetic sugar analogues with a very broad spectrum of activities, including those against the most prominent bacterial pathogens, like *P. aeruginosa* or *S. aureus.* In a series of studies, we have demonstrated that one of the synthetic iminosugars, PDIA (beta-1-C-propyl-1,4-dideoxy-1,4-imino-L-arabinitol), possesses the ability to suppress biofilm production by different pathogenic bacteria without inhibiting their growth. Thereby, PDIA is able to influence experimental skin infection caused by *S. aureus*. **Methods:** To elucidate molecular mechanisms by which PDIA impedes biofilm formation by *S. aureus*, a transcriptomic study was performed in which a biofilm-producing *S. aureus* strain was grown in the presence of PDIA for 24 and 48 h in comparison to a control without the iminosugar. The RNA was then isolated, converted into cDNA, sequenced, and data analysis was performed. **Results:** It appeared that PDIA caused the down-regulation of many bacteriophage genes responsible for the processes of bacterial cell lysis, and some genes responsible for cell wall degradation were also down-regulated. Among the 25 most upregulated genes were those representing the phosphotransferase system (PTS), which is required for carbohydrate uptake and control of carbon metabolism. The ranking of the most significant down-regulated genes after 24 h exposure to PDIA shows that they predominantly coded for both the synthesis and lysis of the peptidoglycan. **Conclusions:** We have shown here that the influence of PDIA on the expression of *S. aureus* genes is broad and affects many genes encoding metabolism and ribosomes.

## 1. Introduction

*Staphylococcus aureus* is a leading cause of death by infections worldwide and the most important pathogen related to hospital-acquired infections, including bloodstream infections [1]. Treatment of *S. aureus* infections is difficult due to widespread antibiotic resistance, which is also related to sessile life in biofilms. Thus, the formation of biofilm is an important way in which *S. aureus* maintains an infection. Staphylococcal biofilms can be formed on abiotic material of indwelling medical devices, as well as on tissue surfaces, such as on heart valves in the case of endocarditis [2]. The main role of biofilm formation during infection is to protect the bacteria from phagocyte attacks [3].

Biofilm formation develops in three main stages: adhesion, maturation/proliferation, and detachment/dispersal. Initial attachment or adhesion occurs to human matrix proteins via cell-wall anchored and other surface proteins, many of which belong to the MSCRAMM family, i.e., microbial surface components recognizing adhesive matrix molecules [4]. In the second stage, for example, during infection, cells divide and produce a biofilm matrix that connects cells. In *S. aureus*, the matrix consists of poly-N-acetylglucosamine (PNAG), also referred to as the polysaccharide intercellular adhesion (PIA), extracellular DNA, teichoic acids, and proteins [5].

Iminosugars are natural or synthetic sugar analogues with an amino function in place of the endocyclic oxygen of the corresponding carbohydrate [6]. They exert their biological effects primarily through inhibition of carbohydrate-processing enzymes such as glycosidases and glycosyltransferases, a mechanism that may also underlie their anti-biofilm properties [7]. Several therapeutic applications of the iminosugars have been proposed and evaluated, but without significant outcomes. Antimicrobial properties of the iminosugars have also been studied, and, indeed, they displayed antiviral [8] and antibacterial inhibitory activities against the most prominent bacterial pathogens like *P. aeruginosa* or *S. aureus* [9,10]. It also appeared that some iminosugars could inhibit biofilm production by bacteria. The most prominent example of this activity is inhibition of *Streptococcus mutans* adherence to dental surfaces by natural 1-deoxynojirimycin from mulberry leaves [11].

Our group studied both inhibitory and anti-biofilm properties of several iminosugars against *P. aeruginosa* and found that the observed compounds inhibited synthesis of the early biofilm but possessed no growth inhibitory properties [12]. Among the evaluated iminosugars, one compound—beta-1-C-propyl-1,4-dideoxy-1,4-imino-L-arabinitol (PDIA)—was selected for further testing (the chemical structure of PDIA is shown in Figure S1). PDIA was subsequently evaluated against 30 clinical strains of major human pathogens, including both Gram-positive and Gram-negative bacteria. While it did not inhibit planktonic growth, it exhibited strong inhibitory activity against early biofilm formation [13]. Recently, the ability of PDIA to interfere with experimental subcutaneous *S. aureus* infection in mice was also demonstrated by us in vivo [14].

While the anti-biofilm activity of PDIA has been demonstrated in vitro and in vivo, the molecular mechanisms underlying this effect remain unclear. Therefore, the aim of this study was to elucidate the transcriptomic response of a biofilm-producing *S. aureus* strain to PDIA treatment, in order to better understand the pathways and genes involved in its anti-biofilm action.

## 2. Results

### 2.1. Analysis of Biofilm Structure by SEM

**Figure 1:**
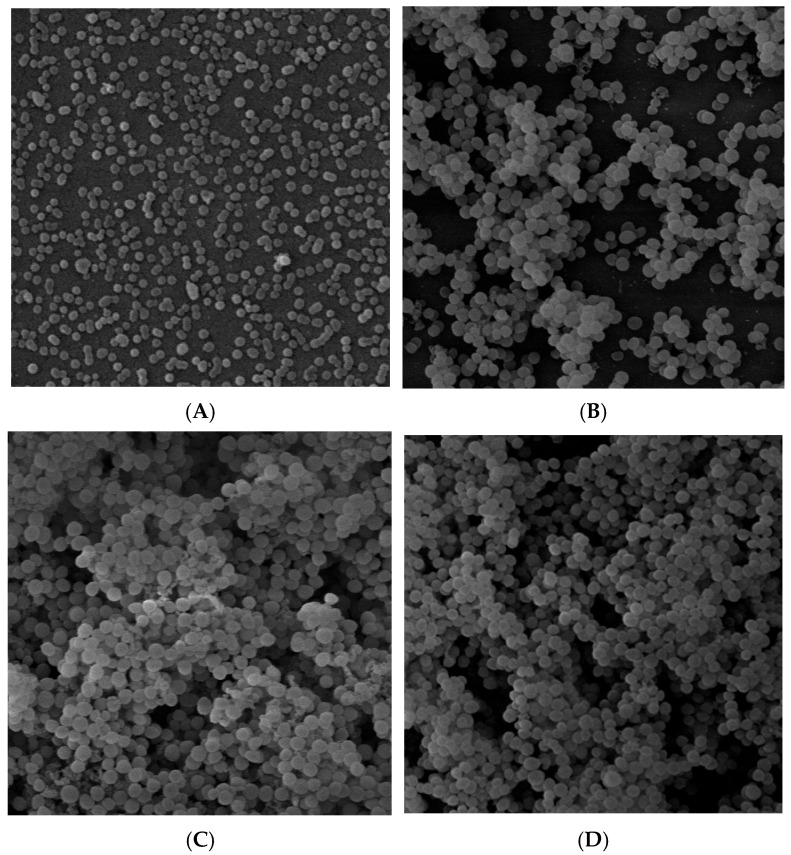
Scanning electron microscopy (SEM) of *S. aureus* 48 biofilms. (**A**): early (24 h) biofilm treated with PDIA; (**B**): untreated early (24 h) biofilm—control; (**C**): mature (48 h) biofilm treated with PDIA; (**D**): untreated mature (48 h) biofilm—control. Densely packed bacterial cells surrounded by an extracellular matrix are visible. Magnification 5000×.

The SEM (scanning electron microscopy) images illustrating the effect of PDIA on the morphology of early and mature biofilms of *S. aureus* 48 are shown in Figure 1. Treatment of *S. aureus* 48 cultures with PDIA resulted in looser biofilm structures, especially in early biofilm, compared to the control.

### 2.2. Transcriptomic Analysis of PDIA-Induced Gene Expression Changes

A total of twelve samples (six per control and six per experimental group) were subjected to RNA-seq. Illumina sequencing yielded approximately 588.39 million raw reads for the control group and 301.77 million raw reads for the experimental group. The average yield was 7.21 and 7.59 gigabases for the control and experimental groups, respectively. A phred score Q30 of 94.48% was achieved for the control group, while this value reached 92.18% for the experimental group. The established type of strain *S. aureus* subsp. *aureus* NCTC 8325 (NCBI RefSeq Assembly GCF_000013425.1) was used as the reference genome in this study. After eliminating low-quality reads with multiple N, reads shorter than 20 bp, and removing sequences encoding rRNA, a total of 293.92 million (142.92 million raw reads for the control group and 150.36 million raw reads for the experimental group) qualified mRNA sequence reads were mapped to the *S. staphylococcus* genome. The average percentage of reads successfully mapped to a reference genome was 63.0 (±11.1). Differentially expressed genes (DEGs) were identified at two time points (24 and 48 h) to reveal transcriptomic differences between the iminosugar-exposed and control groups at *p* ≤ 0.05.

**Figure 2:**
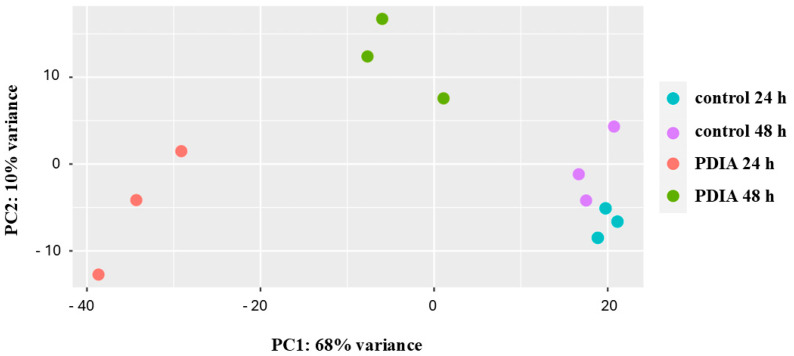
Principal component analysis (PCA) of the general transcriptome characteristics. Samples representing different groups are represented by different colored dots.

First, principal component analysis (PCA) was used to visualize the global differences in gene expression between all samples (Figure 2). It was found that the control samples were grouped independently of the maturity of the biofilm. The samples exposed to PDIA and collected at different times were grouped independently. It is worth noting that PDIA samples representing the mature biofilm were closer to the control samples than the samples collected at 24 h.

In the early biofilm, 1266 downregulated DEGs were identified, of which 716 were significantly downregulated (*p* < 0.05). The results showed that at this stage of biofilm growth, 1397 genes were upregulated and 767 DEGs were significantly upregulated (*p* < 0.05). In the mature biofilm, only 1243 DEGs were identified, of which 316 were significantly downregulated (*p* < 0.05). At this stage, 1353 upregulated DEGs were identified; among them, 289 DEGs showed significant upregulation (*p* < 0.05). A volcano map was used to visualize the overall distribution of DEGs in both groups (Figure S2).

The genes that were statistically significantly downregulated or upregulated in response to PDIA treatment in early biofilm are listed in Table 1 and Table 2, respectively. Many of the down-regulated genes are from bacteriophages (11 genes) and are responsible for the processes of bacterial cell lysis, and some genes responsible for cell wall degradation are also down-regulated. All bacteriophage genes are located next to each other on the chromosome (from SAOUHSC_01519 to SAOUHSC_01539). Genes that are responsible for the degradation of cell structures are located in the immediate vicinity of their functional partners from the phage. All these genes show a similar degree of down-regulation (log2_Estimated_FoldChange from 3.72 to 4.86, mean value of 4.36).

Among the 25 genes that showed the highest upregulation, 11 were genes responsible for tRNA synthesis. The degree of upregulation was relatively variable, as the highest fold change was 11.92 (tRNA-Gly) and the lowest was 4.35 (tRNA-Tyr). The second group of genes was those related to bacteriophages. Most of them represented bacteriophage phiETA, and all of them were upregulated more than four times. Other genes that showed upregulation were responsible for the phosphotransferase system (PTS) (SAOUHSC_02449, SAOUHSC_02450, SAOUHSC_02452) and membrane function (SAOUHSC_01761a and SAOUHSC_02873).

The genes that were statistically significantly downregulated or upregulated in response to PDIA treatment in mature biofilm are listed in Table 3 and Table 4, respectively. Most of the genes that were downregulated in the mature biofilm are the same as those for the early biofilm. Among the phage-derived genes, there were two genes that were downregulated in the early biofilm but not in the mature biofilm (SAOUHSC_01519 and SAOUHSC_01520). Genes responsible for the degradation of cell structures were also downregulated at 48 h. However, the degree of regulation was lower than in the early biofilm (log2_Estimated_FoldChange of 1.76 to 2.70, mean value of 2.17). Genes downregulated in the mature biofilm include those encoding formate C-acetyltransferase (pflB, SAOUHSC_00187) and pyruvate formate lyase 1 activating enzyme (pflA, SAOUHSC_00188). Both showed a relatively high fold change, 2.53 and 2.79, respectively. The genes that showed the strongest upregulation in mature biofilm were the genes encoding azoreductase (SAOUHSC_00173), which was 3.14-fold higher than in the control group, and the gene encoding acylCoA:acetate/3-ketoacid CoA transferase (SAOUHSC_00199) showed upregulation at the 3.01 level.

Among the 25 most upregulated genes were seven representing the phosphotransferase system (PTS), which is required for carbohydrate uptake and control of carbon metabolism. These genes, encoding beta-galactosidase, transporter subunit IIBC, tagatose-1,6-diphosphate aldolase and kinase, and galactose-6-phosphate isomerase, were located sequentially on the chromosome (SAOUHSC_02449-SAOUHSC_02455). In contrast to the early biofilm, the genes belonging to the PTS showed a lower fold change value, which was not higher than 2.63 (SAOUHSC_02450). For the mature biofilm, this value was about 4.0. In contrast to the early biofilm, there were no tRNA-encoding genes among the most upregulated genes after 48 h.

In addition, the DEGs common to the early and mature biofilm were summarized (Table S1). The list contains 52 genes, 45 of which showed the same direct changes. Twenty DEGs were upregulated and 45 were downregulated. The remaining five showed a different direction of change. In general, DEGs common for both time points showed relatively small log2 fold change (below 2.84). Tables S2 and S3 show the DEGs that were unique to the early and mature biofilm, respectively. There were more unique DEGs in the early biofilm (283 DEGs) than in the mature biofilm (175 DEGs). In the early biofilm, the most upregulated genes encode tRNAs, while the downregulated genes are responsible for cell wall structure and related to bacteriophages, among other functions. In the mature biofilm, the most upregulated and downregulated genes code for different proteins.

A comparison of the DEGs of the early and mature biofilms treated with PDIA confirms previous observations that PDIA treatment led to a downregulation of genes responsible for cell wall degradation (e.g., delta-hemolysin and peptidoglycan hydrolase) in the early biofilm (Table S4). At the same time point, the genes responsible for tRNA synthesis were upregulated. The highest upregulation was found for the tRNA-Gly gene (log2_Estimated_FoldChange 7.14).

### 2.3. GO Term Enrichment Analysis of DEGs

The analysis of the functional enrichment of DEGs between the control group and the PDIA-treated groups was based on GO (Gene Ontology) and KEGG (Kyoto Encyclopedia of Genes and Genomes) databases. According to the GO functions, all DEGs were divided into three categories: biological processes (BPs), cellular components (CCs), and molecular functions (MFs). The enrichment analysis (*p* ≤ 0.05) in early biofilm characterized the GO terms as follows (Figure S3). The BP-related DEGs (427 GO terms) were involved in metabolic (52.6%) and cellular processes (47.4%). The CC-related DEGs (36 GO terms) were involved in the cellular anatomical entity (72.7%) and protein-containing complex (27.3%). The MF-related DEGs (238 GO terms) were mainly responsible for transporter activity (42.9%), catalytic activity (28.6%), structural molecule activity (14.3%), and ATP-dependent activity (14.3%).

Whereas the enrichment analysis (*p* ≤ 0.05) of mature biofilm characterized GO terms as follows (Figure S3). The BP-related DEGs (306 GO terms) were involved in metabolic process (46.4%), cellular processes (46.4%), and localization (7.2%). The CC-related DEGs (31 GO terms) were involved in cellular anatomical entity (72.7%) and protein-containing complex (27.3%). The MF-related DEGs (172 GO terms) were mainly responsible for transporter activity (45.8%), catalytic activity (33.3%), binding (16.7%), and structural molecule activity (14.3%)

**Figure 3:**
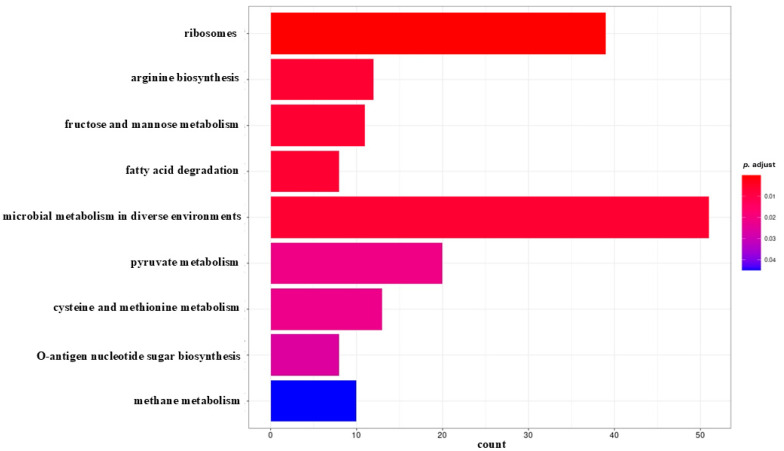
Bar graph showing statistically significant KEGG identification results for the number of genes differentially expressed in the mature biofilm of *S. aureus* compared between the control culture and the culture in the presence of PDIA iminosugar. In the graph, each metabolic/functional pathway is represented by a bar whose length corresponds to the number of genes differentially expressed in that particular pathway. The color of the bar indicates the statistical significance of a particular metabolic pathway, which is determined by the *p*-value.

No significant metabolic pathway was identified for the early biofilm. The results of KEGG analysis showed that DEGs identified in the mature biofilm (Figure 3) were significantly enriched in microbial metabolism in different environments, ribosomes, pyruvate metabolism, cysteine and methionine metabolism, arginine biosynthesis, fructose and mannose metabolism, methane metabolism, fatty acid degradation, and O-antigen nucleotide sugar biosynthesis. Most DEGs (39) with relatively high probability, defined by the adjusted *p*-value, were assigned to the ribosomal pathway. The highest number of DEGs (51) was identified in the microbial metabolism pathway in different environments.

### 2.4. qPCR Validation of RNA-Seq Results

**Figure 4:**
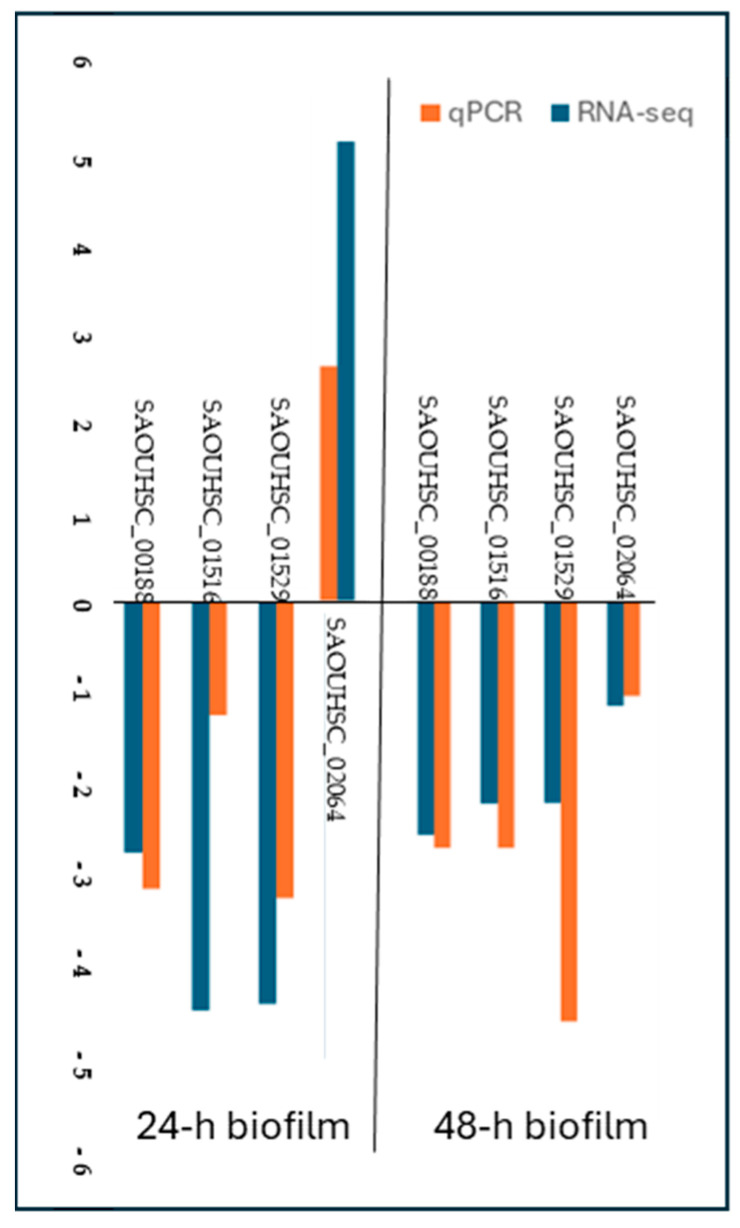
Validation of DEGs in *S. aureus* treated with PDIA iminosugar after 24 and 48 h. Validation was performed using qPCR with normalization using 16S rRNA gene. SAOUHSC_00188—pyruvate formate-lyase 1 activating enzyme, SAOUHSC_01516—holin protein, SAOUHSC_01529—major tail protein, SAOUHSC_02064—phi ETA orf 25-like protein.

To confirm the data obtained by RNA-seq, the expression quantities of the four DEGs were determined with real-time quantitative PCR (Figure 4). The results of qPCR were in good agreement with the RNA-seq data, indicating that the data from RNAseq were of high quality.

## 3. Discussion

Our previous studies on iminosugars and especially on the PDIA compound demonstrated their inhibitory activity directed toward early biofilm formation without any activity against bacterial growth of a wide variety of Gram-positive and Gram-negative bacteria pathogenic for humans [12,13]. The antibiofilm activity of PDIA was also effective in vivo, as shown in a murine model of the staphylococcal subcutaneous infection [14]. However, the molecular mechanism of the antibiofilm activity of PDIA remained unknown.

Studies on iminosugars’ interactions with enzymes revealed that iminosugars are among the most important glycomimetics reported to date due to their powerful activities as inhibitors of a wide variety of glycosidases and glycosyltransferases [15]. Also, galactose-type iminosugars were reported as inhibitors of galactosylransferases [16]. It had also been demonstrated that glucose-mimicking iminosugars of known antiviral activity inhibit isolated glycoprotein and glycolipid processing enzymes in virus-infected cells [17].

More recently, new natural iminosugars with strong antibiofilm activity were obtained from a probiotic strain of *Lactobacillus paragasseri* MJM60645, which was isolated from the human oral cavity. These compounds showed strong inhibitory activities against *S. mutans* biofilm formation but did not show bactericidal activities against *S. mutans*. One of these structures strongly downregulated the expression levels of genes related to biofilm formation in *S. mutans* [18].

We have demonstrated here that the PDIA effects on *S. aureus* genes were broader and were influenced by many genes coding for metabolism and ribosomes. The ranking of the most significant down-regulated genes after 24 h exposure to PDIA shows that they predominantly coded for both synthesis and lysis of the peptidoglycan. The peptidoglycan of the bacterial cell wall undergoes a permanent turnover during cell growth and differentiation. In *S. aureus*, the major peptidoglycan hydrolase Atl is required for accurate cell division, daughter cell separation, and autolysis [19]. Importantly, Atl is associated with autolysis processes, e.g., during biofilm formation. Similar activity is attributed to soluble lytic transglycosylase SLT located in *S. aureus* on the prophage region [20]. Moreover, PDIA causes downregulation of bacteriophage genomes encoded enzymes named holins, which, among others, possess predominantly lytic activities, and thus contribute to biofilm formation [21]. It appeared that holin proteins (CidA, probably in conjunction with LrgA) temporally control the timing of cell lysis and DNA release during biofilm development. In our study, *CidA* showed significantly lower expression (log2_Estimated_FoldChange 2.47) in early biofilm treated with PDIA, confirming the possible role of this protein as a regulator in cell lysis processes. In view of these observations, several authors proposed to use isolated holins or even artificial phages to control infections [22,23].

Significant up-regulation of genes under the influence of PDIA in 24 h was mostly directed toward many tRNA genes. *S. aureus* has a large tRNA cluster which contains 27 tRNA genes immediately 3′ to an rRNA operon. Three of the tRNA genes in this *S. aureus* cluster code for special tRNAs used in the synthesis of peptidoglycan [24].

These observations may suggest that, in 24 h, PDIA induces increased tRNA synthesis and phosphotransferase system (PTS). Moreover, different phage genes are also overexpressed, while others show downregulation. Most probably, PDIA interacts differently with various phage genes, which leads to dysregulation of the peptidoglycan and generally cell wall synthesis, which also impairs biofilm formation.

In the later phase of growth, after 48 h, when the biofilm has matured, the bacterial cells under the influence of PDIA increase the synthesis of proteins involved in carbohydrate metabolism. On the other hand, prophage genes are downregulated. Differences in gene expression between the control culture and the PDIA-treated one are less prominent. This is related to the lifestyle of the mature biofilm. At this stage of growth, the biofilm shows an energy-efficient nature. This could be due to a combination of metabolic and regulatory adaptations, including anaerobic adaptation and reduced expression of virulence-related genes. This multi-layered regulation ensures the survival of *S. aureus* in its biofilm state and enables resilience to external stress factors. In contrast, gene expression is particularly dynamic in the early stages of *S. aureus* biofilm formation due to several key processes that facilitate adhesion, structural integrity, and biofilm formation.

It is of interest that PDIA, at least at the used concentration, does not phenotypically impair *S. aureus* cell division, although staphylococcal cells under the influence of PDIA may have alterations in their cell wall structure. We have not tested such a possibility, but we may speculate that an in vivo curative effect of PDIA on staphylococcal infection may be related not only to inhibition of biofilm formation, but also depressed production of virulence factors, like delta-hemolysin.

The weak point of our studies is that only one concentration of PDIA has been tested against one clinically relevant *S. aureus* with strong biofilm production. Still, the obtained results are highly suggestive of a PDIA activity directed toward bacterial and phage genome genes involved in coding for the synthesis of proteins and polysaccharide macromolecules responsible for building the cell wall and biofilm formation.

The obtained evidence complements the knowledge of the response of *S. aureus* to PDIA iminosugar, providing, together with phenotypic data and in vivo observations, a basis for the development of a highly effective inhibitor for the elimination of *S. aureus* from medical device surfaces and/or the treatment of staphylococcal infections.

## 4. Methods

### 4.1. Bacterial Strain and Growth Conditions

The strain of *S. aureus* 48 was chosen for the experiment based on its demonstrated high biofilm-forming proficiency and the biofilm susceptibility to the inhibitory properties of PDIA iminosugar [13]. This strain is an integral component of the bacterial strains’ repository at the Department of Microbiology, Jagiellonian University Medical College. It was isolated from a patient suffering from chronic otitis media, and its taxonomic identity was verified through mass spectrometry (MALDI-TOF MS Biotyper, Bruker Scientific LLC, Billerica, MA, USA) following the manufacturer’s guidelines [25].

The bacterial inoculum was prepared from the frozen pure culture by incubating the glass beads coated with bacteria in 10 mL of TSB broth (Becton Dickinson, Franklin Lakes NJ, USA) at 37 °C for 24 h. Then, to ensure the purity of the strain, 10 µL aliquots of the culture were streaked over surfaces of Columbia Agar (Biomaxima, Lublin, Poland) and incubated as before. Three passages were made in the same manner to obtain bacterial populations of high viability. Finally, a standardized bacterial suspension was made by transferring 1 µL of the 24-h broth culture to 9 mL of saline, vigorous mixing, and adjusting the optical density to 0.5 on the McFarland scale using a densitometer. This density was adjusted to approximately 1.0 × 10^7^ CFU/mL, as previously controlled with a standard serial dilution method. Such a bacterial stock suspension was used in biofilm production experiments.

### 4.2. Iminosugar

The iminosugar derivative under investigation (PDIA beta-1-C-propyl-1,4-dideoxy-1,4-imino-L-arabinitol) was synthesized at the Institute of Organic and Analytical Chemistry, University of Orleans, France. Our prior research detailed its chemical structure [13]. In the experiments, a solution of iminosugar at a concentration of 0.9 mM was employed. This PDIA concentration was chosen as optimal in a pilot study arranged before studies on in vitro activity.

### 4.3. Biofilm Assay on Polystyrene Plate by Scanning Electron Microscopy (SEM)

In order to show the effect of the tested PDIA iminosugar on both early (24 h) and mature (48 h) biofilms, we analyzed their morphology using scanning electron microscopy (SEM). For this purpose, the glass plates (1 mm thick and 9 mm in diameter) were sterilized and placed into the well of a 12-well plate for biofilm formation. Aliquots (1 mL) of bacterial cell suspensions were seeded into eight wells: four to check biofilm formation under the influence of PDIA, and four were used as controls. To measure the effect of PDIA on the early biofilm, the plate was incubated at 37 °C for 4 h. Then, 200 uL of the bacterial culture was pipetted out of each well, and 200 uL of the PDIA at a final concentration of 0.9 mM was added. A fresh TSB medium *w*/*o* test substance was added to the control wells. The effect of PDIA on mature biofilm formation was measured in the same way as for early biofilm; however, before adding PDIA at a concentration of 0.9 mM, the wells with bacterial cultures were incubated for 24 h. After incubation, the wells were gently washed three times with PBS to remove non-adherent bacteria. The adherent bacteria were fixed and dehydrated. After being fixed with 2.5% glutaraldehyde for 3 h at 4 °C, the surface was rinsed three times with PBS. The sample was dehydrated through 50% ethanol for 10 min each at room temperature. After critical-point drying and 1200 bar pressure at 40 °C, the sample was examined using SEM (Jeol JSM-5410, Akishima, Japan). To ensure reproducibility of results and exclude technical artifacts, SEM analysis was carried out in five replicates, each based on a separate well.

### 4.4. Experimental Design

The effect of PDIA on the production of early (24 h) and mature (48 h) biofilm by *S. aureus* 48 strain was assessed using a 96-well plate model. Wells were filled with 20 µL of freshly prepared suspension of the tested *S. aureus* 48 strain containing 1 × 10^7^ CFU/mL and 180 µL of TSB broth (Becton Dickinson). Six wells were used for biofilm formation under the influence of the PDIA (experimental group), six wells for biofilm formation in the control group, and the next two wells for measuring the number of viable bacteria.

The plates were incubated at 37 °C under aerobic conditions for 4 h to allow initial adhesion. In the next step, 180 µL of the bacterial culture was pipetted out of each experimental well, and 180 µL of the PDIA at a final concentration of 0.9 mM was added. In the control wells, an equivalent volume of fresh TSB medium without PDIA was added. The plates were gently rotated to distribute the iminosugar and incubated for 24 h at 37 °C in aerobic conditions. For RNA isolation, the early biofilm was carefully scraped from the well surface using a sterile cell scraper and transferred into microcentrifuge tubes containing 500 µL of RNAlater (QIAGEN, Austin, TX, USA). The samples were then vortexed briefly to resuspend the biofilm.

The effect of PDIA on mature biofilm was assessed using the same methodology. However, prior to PDIA treatment, bacterial cultures were incubated for 24 h to allow biofilm maturation. PDIA at 0.9 mM was then added, and biofilm samples for RNA isolation were collected at 48 h post-inoculation.

### 4.5. RNA Isolation

The total RNA from approximately 25 mg of *S. aureus* bacterial culture was isolated using Bead-Beat Total RNA Mini (A&A Biotechnology, Gdańsk, Poland). To lyse cells, 790 µL of fenozol supplemented with 10 µL of lysostaphin solution (15 U/µL) was added, mixed by pipetting, and transferred into a bead-beating tube. Samples were homogenized at maximum speed in 20 s on/60 s off intervals for 3 min total (Uniequip, Planegg, Germany). During the rest period, samples were cooled on ice. Next, samples were incubated for 5 min at 50 C, after which 200 µL of chloroform was added, mixed, and left for 3 min at room temperature. Subsequently, samples were centrifuged for 10 min at 12,000 rpm. Supernatant was then transferred into a new tube, mixed with 250 µL of isopropanol, transferred onto a minicolumn, and centrifuged for 1 min at 12,000 rpm. After three washing steps with A1 wash solution, the minicolumn was left to dry, and RNA was eluted with 60 µL of RNAse-free water. The content of RNA was measured with a Qubit 3 fluorometer (Thermo Fisher Scientific, Waltham, MA, USA). RNA purity was additionally assessed with Nanodrop 8000 (Thermo Fisher Scientific, Waltham, MA, USA). RNA integrity was evaluated using the Bioanalyzer 2100 (Agilent, Santa Clara, CA, USA). Until subsequent analysis, the isolated RNA was kept at −80 °C.

### 4.6. Sequencing

Sequencing was carried out by Macrogen Europe following TruSeq Stranded Total RNA Reference Guide (1000000040499 v00, October 2017). rRNA was depleted with NEBNext rRNA Depletion (Bacteria). The TruSeq Stranded Total RNA Library Prep Gold Kit (Illumina, San Diego, CA, USA) was used to prepare libraries. Sequencing was conducted using the Hiseq 2500 platform (Illumina, San Diego, CA, USA) in pair-end mode.

### 4.7. Data Analysis

The first step of the analysis involved adapter trimming using Cutadapt (ver. 4.9) tool [26]. Filtering was performed using standard parameters, using a quality threshold of q = 25 and a minimum read length of m = 20. Quality reports for the sequencing data were generated with FastQC (ver. 0.12.0). Next, the reads were mapped to the ASM1342v1 reference genome of *S. aureus* (NCBI RefSeq assembly GCF_000013425.1) using HISAT2 (ver. 2.2.1) [27]. The mapping process was performed with the --rna-strandness RF option to account for strand-specific RNA libraries. Following alignment, the read counts mapped to individual genes were quantified using HTSeq (ver. 0.9.1) [28], with the --stranded = reverse option to account for the reverse strand-specific nature of the data. Only genes with a cumulative total of at least three mapped reads across all samples were included in the analysis. Gene annotations were assigned based on the corresponding feature description file (feature_table.txt) for the *S. aureus* genome. Additionally, InterProScan (ver. 5.60–92.0) [29] was employed to retrieve Gene Ontology (GO) identifiers for the analyzed genes. To identify differentially expressed genes between defined comparisons, the final results were processed in R using the DESeq2 package [30] (Benjamin-Hochberg padj < 0.05). Enriched GO terms were identified using the topGO package (ver. 2.38.31) [31]. Finally, biological pathways for statistically significant genes with EntrezID identifiers were determined using the clusterProfiler package (ver. 4.12.0) [32] and the KEGG database [33].

### 4.8. Real-Time PCR Validation

Validation of RNA-Seq data via qRT-PCR was conducted to quantify the mRNA transcripts of four randomly selected DEGs. Details of the primers used in the current study are listed in Supplementary Table S5. PCR primers were designed using Primer3web (ver. 4.1.0). The total RNA isolated from *S. aureus* under identical processing conditions to those of the RNA-Seq samples was subjected to cDNA synthesis using a SuperScript VILO cDNA Synthesis Kit (Illumina, USA). The PCR reaction was performed in a QuantStudio™ 3 thermocycler (Applied Biosystems, Carlsbad, CA, USA), in MicroAmp™ Fast Optical 96-Well Reaction Plates, using Mix SYBR^®^ A RT PCR reagents (A&A Biotechnology, Gdynia, Poland). Each sample was analyzed in triplicate. The resulting data were analyzed using the relative quantification method, with control as the reference sample, whereas 16S rRNA was used as an internal control to normalize the target gene mRNA expression. Relative quantification was calculated using the formula RQ = 2^−ΔCt^, where ΔCt represents the difference between the Ct value of the marker gene in the test sample and that of the same gene in the reference sample [34].

## 5. Conclusions

Iminosugar PDIA causes multiple changes in gene expression in *S. aureus* cells that explain its inhibitory effect on biofilm production. In 24 h, increased tRNA synthesis is observed, and genes coding for the phosphotransferase system (PTS) are activated. At the same time, some bacteriophage genes are overexpressed, and others are depressed. Most probably, PDIA causes deregulation of genes involved in the synthesis and function of the staphylococcal cell wall. Increased tRNA synthesis may represent repair mechanisms by which peptidoglycan synthesis is accelerated. We have not demonstrated a specific mechanism of the biofilm inhibitory activity of PDIA. In 48 h, the bacterial cell under the influence of PDIA tried to save its cell wall structure by increasing the synthesis of proteins involved in carbohydrate metabolism, together with depressing phage genes responsible for cell wall degradation. The gene regulatory effect of PDIA in this phase of bacterial growth is thus less prominent.

While further functional studies are needed to validate specific pathways, our transcriptomic analysis provides a valuable foundation for understanding the complex regulatory effects of PDIA on biofilm-forming *S. aureus*.

## Figures and Tables

**Table 1:** Top 25 significantly downregulated genes (DEGs) in early *S. aureus* biofilm following PDIA exposure.

Gene	log^2^ Fold Change	FDR Adjusted *p*-Value	Name
SAOUHSC_02260	−9.39	3.46 × 10^−279^	delta-hemolysin
SAOUHSC_01532	−5.23	3.80 × 10^−30^	SLT orf 110-like protein
SAOUHSC_01536	−4.86	8.86 × 10^−69^	scaffolding protease
SAOUHSC_01531	−4.83	5.88 × 10^−54^	SLT orf 123-like protein
SAOUHSC_01516	−4.74	8.96 × 10^−33^	holin protein
SAOUHSC_01529	−4.67	1.39 × 10^−57^	major tail protein
SAOUHSC_01451	−4.63	7.64 × 10^−58^	threonine dehydratase
SAOUHSC_01528	−4.62	7.94 × 10^−59^	bacteriophage L54aIg-like domain
SAOUHSC_02969	−4.56	7.74 × 10^−47^	arginine deiminase
SAOUHSC_01519	−4.45	3.99 × 10^−35^	SLT orf 129-like protein
SAOUHSC_01515	−4.39	1.75 × 10^−65^	peptidoglycan hydrolase
SAOUHSC_01539	−4.21	3.33 × 10^−16^	terminase small subunit
SAOUHSC_01521	−4.19	5.90 × 10^−52^	SLT orf 636-like protein
SAOUHSC_01520	−4.19	2.91 × 10^−46^	SLT orf 488-like protein
SAOUHSC_02968	−4.11	2.80 × 10^−46^	ornithine carbamoyltransferase
SAOUHSC_01537	−4.07	1.55 × 10^−41^	HK97 family phage portal protein
SAOUHSC_01452	−4.07	4.61 × 10^−34^	alanine dehydrogenase
SAOUHSC_00533	−4.04	2.96 × 10^−53^	chaperone protein HchA
SAOUHSC_01524	−3.96	6.66 × 10^−40^	holin-like protein
SAOUHSC_01538	−3.95	1.04 × 10^−39^	phage terminase large subunit
SAOUHSC_00608	−3.81	3.37 × 10^−36^	alcohol dehydrogenase
SAOUHSC_01788	−3.77	1.36 × 10^−59^	threonyl-tRNA synthetase
SAOUHSC_01278	−3.74	2.45 × 10^−68^	aerobic glycerol-3-phosphate dehydrogenase
SAOUHSC_01525	−3.73	4.46 × 10^−58^	phage tail tape meausure protein
SAOUHSC_01523	−3.72	1.54 × 10^−36^	SLT orf 527-like protein

**Table 2:** Top 25 significantly upregulated genes (DEGs) in early *S. aureus* biofilm following PDIA Exposure.

Gene	log^2^ Fold Change	FDR Adjusted *p*-Value	Name
SAOUHSC_T00021	11.92	2.18 × 10^−5^	tRNA-Gly
SAOUHSC_T00041	7.14	1.75 × 10^−7^	tRNA-Met
SAOUHSC_T00058	6.55	5.79 × 10^−5^	tRNA-Tyr
SAOUHSC_T00053	6.36	3.97 × 10^−5^	tRNA-Thr
SAOUHSC_T00035	5.5	3.48 × 10^−6^	tRNA-Lys
SAOUHSC_02064	5.3	2.04 × 10^−7^	phi ETA orf 25-like protein
SAOUHSC_T00051	5.25	1.80 × 10^−14^	tRNA-Ser
SAOUHSC_02050	5.16	0.005511766	terminase small subunit
SAOUHSC_T00016	4.96	0.012930175	tRNA-Gln
SAOUHSC_T00014	4.75	3.23 × 10^−16^	tRNA-Asp
SAOUHSC_T00022	4.7	0.005922209	tRNA-Gly
SAOUHSC_T00056	4.67	0.024895614	tRNA-Trp
SAOUHSC_02217	4.59	3.15 × 10^−53^	phi ETA orf 22-like protein
SAOUHSC_02216	4.53	2.31 × 10^−66^	phage DnaC-like protein
SAOUHSC_02080	4.4	5.02 × 10^−57^	bacteriophage L54a antirepressor
SAOUHSC_02078	4.35	3.57 × 10^−40^	phi PV83 orf 10-like protein
SAOUHSC_T00057	4.35	0.00974457	tRNA-Tyr
SAOUHSC_02219	4.33	7.93 × 10^−42^	phi ETA orf 20-like protein
SAOUHSC_01761a	4.24	3.60 × 10^−30^	membrane protein
SAOUHSC_02873	4.21	5.93 × 10^−41^	cation transporter E1-E2 family ATPase
SAOUHSC_02213	4.15	1.59 × 10^−15^	phi ETA orf 25-like protein
SAOUHSC_02450	4.14	7.53 × 10^−80^	PTS system lactose-specific transporter
SAOUHSC_00097	4.06	7.82 × 10^−42^	purine nucleoside phosphorylase
SAOUHSC_02449	4.04	5.44 × 10^−56^	6-phospho-beta-galactosidase
SAOUHSC_02452	3.97	2.45 × 10^−66^	tagatose 1,6-diphosphate aldolase

**Table 3:** Top 25 significantly downregulated genes (DEGs) in mature *S. aureus* biofilm following PDIA exposure.

Gene	log^2^ Fold Change	FDR Adjusted *p*-Value	Name
SAOUHSC_00188	−2.79	2.73 × 10^−17^	pyruvate formate-lyase 1 activating enzyme
SAOUHSC_01528	−2.7	5.21 × 10^−6^	bacteriophage L54aIg-like domain
SAOUHSC_01515	−2.57	0.008840928	peptidoglycan hydrolase
SAOUHSC_00187	−2.53	8.45 × 10^−13^	formate acetyltransferase
SAOUHSC_01516	−2.41	0.000287074	holin protein
SAOUHSC_01529	−2.4	3.35 × 10^−5^	major tail protein
SAOUHSC_01531	−2.33	4.59 × 10^−5^	SLT orf 123-like protein
SAOUHSC_01536	−2.33	0.000101285	scaffolding protease
SAOUHSC_02260	−2.3	4.58 × 10^−7^	delta-hemolysin
SAOUHSC_01532	−2.19	0.003732062	SLT orf 110-like protein
SAOUHSC_02958	−2.18	5.83 × 10^−6^	alkaline phosphatase III
SAOUHSC_01537	−2.08	0.000446571	HK97 family phage portal protein
SAOUHSC_01521	−2.07	0.038669542	SLT orf 636-like protein
SAOUHSC_01525	−2.01	0.000109535	phage tail tape meausure protein
SAOUHSC_01386	−1.99	0.001324074	phosphate ABC transporter permease
SAOUHSC_01566	−1.97	8.77 × 10^−5^	phi APSE P51-like protein
SAOUHSC_00142	−1.9	7.03 × 10^−10^	formate dehydrogenase
SAOUHSC_01538	−1.88	0.001015173	phage terminase large subunit
SAOUHSC_01570	−1.87	0.000306708	PVL orf 37-like protein
SAOUHSC_01539	−1.83	0.011205785	terminase small subunit
SAOUHSC_01523	−1.81	0.00306758	SLT orf 527-like protein
SAOUHSC_02606	−1.79	9.95 × 10^−6^	imidazolonepropionase
SAOUHSC_00608	−1.76	2.94 × 10^−8^	alcohol dehydrogenase
SAOUHSC_01524	−1.76	0.002256919	holin-like protein
SAOUHSC_00120	−1.75	1.56 × 10^−20^	UDP-N-acetylglucosamine 2-epimerase

**Table 4:** Top 25 significantly upregulated genes (DEGs) in mature *S. aureus* biofilm following PDIA exposure.

Gene	log^2^ Fold Change	FDR Adjusted *p*-Value	Name
SAOUHSC_00173	3.14	1.61 × 10^−26^	azoreductase
SAOUHSC_00199	3.01	3.42 × 10^−12^	acyl CoA:acetate/3-ketoacid CoA transferase
SAOUHSC_02450	2.63	1.06 × 10^−34^	PTS system lactose-specific transporter subunit IIBC
SAOUHSC_02452	2.62	1.43 × 10^−34^	tagatose 1,6-diphosphate aldolase
SAOUHSC_02455	2.45	1.34 × 10^−17^	galactose-6-phosphate isomerase subunit LacA
SAOUHSC_02453	2.4	5.20 × 10^−20^	tagatose-6-phosphate kinase
SAOUHSC_02451	2.4	6.33 × 10^−16^	PTS system lactose-specific transporter subunit IIA
SAOUHSC_02662	2.4	1.04 × 10^−10^	PTS system sucrose-specific transporter subunit IIBC
SAOUHSC_01761a	2.36	2.57 × 10^−5^	membrane protein
SAOUHSC_00132	2.31	0.02681717	aldehyde dehydrogenase
SAOUHSC_02729	2.25	1.13 × 10^−10^	amino acid ABC transporter-like protein
SAOUHSC_02454	2.23	1.99 × 10^−12^	galactose-6-phosphate isomerase subunit LacB
SAOUHSC_02449	2.17	1.97 × 10^−20^	6-phospho-beta-galactosidase
SAOUHSC_01991	2.17	3.72 × 10^−6^	ABC transporter permease
SAOUHSC_01012	2.17	0.036879081	phosphoribosylformylglycinamidine synthase I
SAOUHSC_01327	1.96	0.000190593	catalase
SAOUHSC_00992	1.93	7.28 × 10^−8^	MarR family transcriptional regulator
SAOUHSC_01691	1.89	2.31 × 10^−6^	DNA internalization-related competence protein
SAOUHSC_00624	1.84	1.13 × 10^−5^	integrase/recombinase
SAOUHSC_02270	1.84	0.032778238	ammonium transporter
SAOUHSC_00871	1.83	0.009866049	D-alanine-poly(phosphoribitol) ligase subunit 2
SAOUHSC_00195	1.82	0.0003095	acetyl-CoA acetyltransferase
SAOUHSC_00221	1.79	0.004285467	alcohol dehydrogenase
SAOUHSC_01990	1.76	0.000106789	amino acid ABC transporter ATP-binding protein
SAOUHSC_00899	1.74	7.79 × 10^−5^	argininosuccinate synthase

## Data Availability

Raw data of DNA sequences were deposited in the NCBI SRA database under BioProject nr PRJNA987442. The original contributions presented in this study are included in the article/Supplementary Materials. Further inquiries can be directed to the corresponding author.

## Supplementary Materials

The following supporting information can be downloaded at: https://www.mdpi.com/article/10.3390/antibiotics14070668/s1, Figure S1: Functional comparison of DEGs in the PDIA group compared to the control group in early- and mature biofilm. Functions are organized into three groups, namely Biological processes, Cellular components, and Molecular functions. Figure S2. Volcano graph generated for detected differentially expressed genes in early biofilm (A) and mature biofilm (B) of *S. aureus*, comparing the control culture with the culture in the presence of PDIA iminosugar. Figure S3. Functional comparison of DEGs in the PDIA treated-group compared to control group in early- and mature biofilm. Table S1. List of common differentially expressed genes (DEG) identified in both the 24 h and 48 h groups. Table S2. List of differentially expressed genes (DEG) uniquely identified in the 24 h group. Table S3. List of differentially expressed genes (DEG) uniquely identified in the 48 h group. Table S4. List of differentially expressed genes (DEG) identified in DESeq2 analysis between both exposed groups (24 h vs. 48 h). Table S5. Details of PCR primers used for qPCR.

## Abbreviations

DIA (beta-1-C-propyl-1,4-dideoxy-1,4-imino-L-arabinitol); SEM (scanning electron microscopy); PTS (phosphotransferase system); PCA (principal component analysis); GO (gene ontology); KEGG (Kyoto encyclopedia of genes and genomes); BP (biological processes); CC (cellular components); MF (molecular functions).
