# Supplementary material for: Transcriptomic Analysis of Biofilm Formation Inhibition by PDIA Iminosugar in Staphylococcus aureus
Source: Antibiotics (Basel). 2025 Jul 1;14(7):668. doi: 10.3390/antibiotics14070668 (PMC12291779; doi:10.3390/antibiotics14070668)

Table S1. List of common differentially expressed genes (DEG) identified in both the 24h and 48h groups. Genes were selected based on a false discovery rate (FDR) < 0.05 and an absolute $\log_2$ fold change ($|\log_2 FC|$) > 0.5. The list includes genes with opposite regulation directions between the time points. Genes are sorted by their $\log_2$ fold change at 24 hours ("log2_Estimated_FoldChange_24h").

Table S1. List of common differentially expressed genes (DEG) identified in both the 24h and 48h groups. Genes were selected based on a false discovery rate (FDR) < 0.05 and an absolute $\log_2$ fold change ($|\log_2 FC|$) > 0.5. The list includes

Gene	FDR_adjusted_p_value_24h	log2_Estimated_FoldChange_24h	FDR_adjusted_p_value_48h	log2_Estimated_FoldChange_48h	name
SAOUHSC_00914	0.002962976	2.84	0.018215808	1.07	2-isopropylmalate synthase
SAOUHSC_02270	0.000111918	2.56	0.032778237	1.84	ammonium transporter
SAOUHSC_01939	0.000172608	1.67	0.018215808	1	serine protease SplC
SAOUHSC_02564	0.000454898	1.66	0.000710049	-0.84	urease accessory protein UreG
SAOUHSC_02563	0.002697963	1.5	0.000542746	-0.92	urease accessory protein UreF
SAOUHSC_02963	0.001991987	1.11	0.000755154	1.25	clumping factor B
SAOUHSC_02482	0.000301175	0.99	0.000682046	0.9	cobalt transporter ATP-binding subunit
SAOUHSC_00842	0.005728961	0.99	0.034230212	0.51	ABC transporter ATP-binding protein
SAOUHSC_02682	0.004407414	0.89	0.035654128	-0.67	uroporphyrin-III C-methyltransferase
SAOUHSC_00329	0.008538538	0.87	0.003511779	0.94	mttA/Hcf106 family protein-like protein
SAOUHSC_01591	0.000885042	0.86	0.000104754	0.76	integrase/recombinase XerD
SAOUHSC_00221	0.003427661	0.82	0.004285466	1.79	alcohol dehydrogenase
SAOUHSC_02481	0.002129274	0.81	0.000458487	0.78	cobalt transport protein
SAOUHSC_01684	0.001846846	0.78	0.044386517	0.53	heat shock protein GrpE
SAOUHSC_00549	0.001097009	0.76	0.010402135	0.6	GTP cyclohydrolase
SAOUHSC_01194	0.000580807	0.71	0.003962924	0.7	ATP-dependent DNA helicase RecG
SAOUHSC_02754	0.049648034	0.64	0.014688517	-0.71	ABC transporter ATP-binding protein
SAOUHSC_01617	0.002397770	0.62	0.011043178	0.86	arginine repressor
SAOUHSC_01102	0.005014181	0.59	0.003658198	0.67	excinuclease ABC subunit C
SAOUHSC_02834	0.002705123	0.58	0.003732061	0.81	sortase
SAOUHSC_02696	0.011951046	0.57	0.024924377	0.6	methicillin resistance determinant protein FmhA
SAOUHSC_01599	0.008470756	0.52	0.002492306	0.71	glucose-6-phosphate 1-dehydrogenase
SAOUHSC_01043	0.025755889	0.47	0.004087899	-0.79	dihydrolipoamide dehydrogenase
SAOUHSC_01261	0.012466264	0.45	0.000917048	0.77	competence-damage inducible protein cinA
SAOUHSC_00785	0.027434913	0.45	0.023622877	0.62	thioredoxin reductase
SAOUHSC_01829	0.020633445	-0.39	0.000209243	-0.7	30S ribosomal protein S4
SAOUHSC_00527	0.037770330	-0.46	0.013459620	-1.46	30S ribosomal protein S12
SAOUHSC_02114	0.002616840	-0.72	0.024924377	-0.52	lipid kinase
SAOUHSC_02848	0.048038433	-0.72	0.013626865	-0.81	PTS system glucose-specific transporter subunit IIABC
SAOUHSC_02855	0.005215585	-0.77	0.031038961	-0.86	LysM domain-containing protein
SAOUHSC_00209	0.014460812	-0.79	0.036687718	-0.7	PTS system glucose-specific transporter subunit IIBC
SAOUHSC_01909	0.007184333	-0.81	0.004917379	-0.72	S-adenosylmethionine synthetase
SAOUHSC_02909	0.021192266	-0.81	0.000836153	-0.91	dihydroorotate dehydrogenase 2
SAOUHSC_01000	0.049084731	-0.81	0.022492440	-0.52	cytochrome c oxidase subunit III
SAOUHSC_00217	0.027471681	-0.85	0.018684024	-0.97	sorbitol dehydrogenase
SAOUHSC_00999	0.034784340	-0.87	0.000142905	-0.87	quinol oxidase subunit IV
SAOUHSC_00708	0.000209231	-0.89	0.021729518	0.9	fructose specific permease
SAOUHSC_01129	0.047640956	-0.89	0.000207442	-1.28	carbamate kinase
SAOUHSC_00530	0.005558395	-0.94	0.014541720	-0.71	elongation factor Tu
SAOUHSC_01771	0.000195711	-0.98	0.044352549	-0.54	glutamate-1-semialdehyde aminotransferase
SAOUHSC_00374	0.000370961	-1.04	0.004288799	-1.35	inosine-5'-monophosphate dehydrogenase
SAOUHSC_00519	0.000409063	-1.06	0.000905906	-0.74	50S ribosomal protein L1
SAOUHSC_02341	0.000536956	-1.06	0.022562633	-0.66	F0F1 ATP synthase subunit beta
SAOUHSC_02353	0.001097039	-1.12	0.048460698	-0.53	uracil phosphoribosyltransferase
SAOUHSC_02352	0.000551107	-1.14	0.043110163	-0.46	UDP-GlcNAc 2-epimerase
SAOUHSC_00926	0.002945855	-1.19	0.005197375	0.85	oligopeptide ABC transporter ATP-binding protein
SAOUHSC_00124	0.011300764	-1.19	0.034888271	-1.22	capsular polysaccharide biosynthesis protein Cap5K
SAOUHSC_02922	0.018601414	-1.22	0.025867481	-0.67	L-lactate dehydrogenase
SAOUHSC_00123	0.004605492	-1.26	0.011912917	-1.23	capsular polysaccharide biosynthesis protein Cap5J
SAOUHSC_00797	0.000538804	-1.3	0.015848629	-1.23	triosephosphate isomerase
SAOUHSC_00871	0.000115294	-1.34	0.009866048	1.83	D-alanine--poly(phosphoribitol) ligase subunit 2
SAOUHSC_02208	0.000724416	-1.84	0.005406380	-1.55	PV83 orf 27-like protein

Table S2. List of differentially expressed genes (DEG) uniquely identified in the 24h group. Genes were selected based on a false discovery rate (FDR) < 0.05 and an absolute $\log_2$ fold change ($|\log_2 FC|$) > 0.5. Only genes not differentially expressed in the 48h group under the same thresholds are included. Genes are sorted by their $\log_2$ fold change at 24 hours ("log2_Estimated_FoldChange").

Gene	FDR_adjusted_p_value	log2_Estimated_FoldChange	name
SAOUHSC_T00058	0.000190263	6.55	tRNA-Tyr
SAOUHSC_T00053	0.000136702	6.36	tRNA-Thr
SAOUHSC_02050	0.011836307	5.16	terminase small subunit
SAOUHSC_T00016	0.025412179	4.96	tRNA-Gln
SAOUHSC_T00022	0.012585874	4.7	tRNA-Gly
SAOUHSC_T00056	0.045536589	4.67	tRNA-Trp
SAOUHSC_T00057	0.019763530	4.35	tRNA-Tyr
SAOUHSC_T00059	0.027422652	4.29	tRNA-Val
SAOUHSC_T00052	0.001614785	3.93	tRNA-Ser
SAOUHSC_02053	0.002005398	3.89	transcriptional activator rinb-like protein
SAOUHSC_T00032	0.000325832	3.69	tRNA-Leu
SAOUHSC_T00046	0.012936268	3.57	tRNA-OTHER
SAOUHSC_00352	0.001198967	2.36	integrase-like protein
SAOUHSC_03005	0.001125920	2.33	intercellular adhesion protein C
SAOUHSC_03014	0.000165837	2.31	ATP phosphoribosyltransferase catalytic subunit
SAOUHSC_00970	0.001455865	2.13	ABC transporter ATP-binding protein
SAOUHSC_00629	0.002448411	1.98	monovalent cation/H+ antiporter subunit E

SAOUHSC_02028	0.002528289	1.88	phiETA ORF57-like protein
SAOUHSC_03004	0.004930025	1.87	intercellular adhesion protein B
SAOUHSC_01367	0.003472656	1.8	anthranilate synthase component II
SAOUHSC_00359	0.000519500	1.75	phosphoglycerate mutase family protein
SAOUHSC_01942	0.002442350	1.74	serine protease SplA
SAOUHSC_00794	0.000497537	1.72	glycolytic operon regulator
SAOUHSC_02207	0.013028512	1.72	phi PVL/orf 52-like protein
SAOUHSC_01938	0.000139815	1.63	serine protease SplD
SAOUHSC_02098	0.000693327	1.58	DNA-binding response regulator VraR
SAOUHSC_00199	0.000363818	1.49	acyl CoA:acetate/3-ketoacid CoA transferase
SAOUHSC_00434	0.000117607	1.46	LysR family transcriptional regulator
SAOUHSC_01366	0.004964251	1.43	anthranilate synthase component I
SAOUHSC_00291	0.004715100	1.41	PfkB family carbohydrate kinase
SAOUHSC_R00016	0.033105977	1.39	5S Ribosomal RNA
SAOUHSC_T00048	0.004312992	1.37	tRNA-Ser
SAOUHSC_02765	0.000171936	1.35	nickel ABC transporter permease
SAOUHSC_02729	0.000325041	1.35	amino acid ABC transporter-like protein
SAOUHSC_02723	0.000161917	1.32	glycerate kinase

SAOUHSC_00008	0.002405389	1.28	histidine ammonia-lyase
SAOUHSC_00232	0.033472061	1.24	murein hydrolase regulator LrgA
SAOUHSC_00386	0.009781011	1.23	superantigen-like protein
SAOUHSC_02764	0.000171361	1.19	peptide ABC transporter ATP-binding protein
SAOUHSC_03036	0.002360548	1.18	ABC transporter ATP-binding protein
SAOUHSC_02329	0.000641625	1.17	hydroxyethylthiazole kinase
SAOUHSC_01903	0.034178542	1.17	camphor resistance protein CrcB
SAOUHSC_02113	0.008784442	1.12	RNA methyltransferase
SAOUHSC_02374	0.000351247	1.08	aminobenzoyl-glutamate utilization protein B
SAOUHSC_00324	0.000245555	1.06	50S ribosomal protein L7 serine acetyltransferase
SAOUHSC_01991	0.014546277	1.04	ABC transporter permease
SAOUHSC_01972	0.003089639	1.03	protein export protein PrsA
SAOUHSC_03045	0.025388003	1.01	cold shock protein
SAOUHSC_00471	0.000222281	0.98	bifunctional N-acetylglucosamine-1-phosphate uridyltransferase/glucosamine-1-phosphate acetyltransferase
SAOUHSC_03023	0.001463923	0.98	lactonase Drp35

SAOUHSC_02542	0.007960647	0.97	molybdopterin biosynthesis protein MoeA
SAOUHSC_02480	0.000173640	0.95	tRNA pseudouridine synthase A
SAOUHSC_03055	0.008697498	0.94	50S ribosomal protein L34
SAOUHSC_03011	0.003310270	0.93	imidazoleglycerol-phosphate dehydratase
SAOUHSC_02328	0.006228514	0.92	thiamine-phosphate pyrophosphorylase
SAOUHSC_00544	0.000189880	0.91	fibrinogen-binding protein SdrC
SAOUHSC_00912	0.000111707	0.89	ATP-dependent Clp protease ATP-binding subunit ClpB
SAOUHSC_00894	0.001530585	0.88	ornithine--oxo-acid transaminase
SAOUHSC_00477	0.001985456	0.88	transcription-repair coupling factor
SAOUHSC_02005	0.004747020	0.87	A/G-specific adenine glycosylase
SAOUHSC_02255	0.034741383	0.87	co-chaperonin GroES
SAOUHSC_03052	0.000136548	0.86	tRNA uridine 5-carboxymethylaminomethyl modification protein GidA
SAOUHSC_00178	0.000145148	0.85	maltose ABC transporter permease
SAOUHSC_03037	0.011813744	0.85	permease
SAOUHSC_01038	0.013295019	0.85	peptide deformylase
SAOUHSC_02988	0.000821471	0.84	accessory Sec system protein Asp1
SAOUHSC_00130	0.010369964	0.83	heme-degrading monooxygenase IsdI

SAOUHSC_01757	0.000708771	0.81	50S ribosomal protein L21
SAOUHSC_00803	0.003478116	0.81	ribonuclease R
SAOUHSC_00902	0.005242100	0.81	signal peptidase IA
SAOUHSC_03019	0.000565758	0.8	ABC transporter ATP-binding protein
SAOUHSC_02315	0.000718100	0.78	DNA-binding response regulator
SAOUHSC_01103	0.002461739	0.77	succinate dehydrogenase cytochrome b-558 subunit
SAOUHSC_00236	0.002722531	0.77	6-phospho-beta-glucosidase
SAOUHSC_01809	0.009316934	0.77	acetyl-CoA carboxylase carboxyltransferase subunit beta
SAOUHSC_02760	0.001123483	0.76	glutamate synthase subunit alpha
SAOUHSC_00454	0.010490923	0.76	DNA polymerase III subunit delta'
SAOUHSC_02610	0.012407023	0.76	formimidoylglutamase
SAOUHSC_02987	0.000397818	0.74	accessory Sec system protein Asp2
SAOUHSC_01808	0.005427326	0.74	acetyl-CoA carboxylase carboxyltransferase subunit alpha
SAOUHSC_00472	0.007365464	0.73	ribose-phosphate pyrophosphokinase
SAOUHSC_02057	0.008309271	0.73	dUTP pyrophosphatase
SAOUHSC_00076	0.042601482	0.73	2.3-diaminopropionate biosynthesis protein SbnB

SAOUHSC_01992	0.001023874	0.71	PTS system transporter subunit IIC domain-containing protein
SAOUHSC_02763	0.015850835	0.71	peptide ABC transporter ATP-binding protein
SAOUHSC_00888	0.000136759	0.69	monovalent cation/H+ antiporter subunit B
SAOUHSC_01755	0.017581379	0.69	50S ribosomal protein L27
SAOUHSC_00475	0.044684759	0.68	peptidyl-tRNA hydrolase
SAOUHSC_00780	0.000175625	0.66	excinuclease ABC subunit A
SAOUHSC_02316	0.001169122	0.66	DEAD-box ATP dependent DNA helicase
SAOUHSC_00445	0.011028272	0.66	recombination protein RecR
SAOUHSC_02859	0.014487314	0.66	hydroxymethylglutaryl-CoA reductase
SAOUHSC_00998	0.018601414	0.66	methicillin resistance protein FmtA
SAOUHSC_01048	0.041675201	0.66	spermidine/putrescine ABC transporter permease
SAOUHSC_01040	0.000521731	0.65	pyruvate dehydrogenase complex. E1 component subunit alpha
SAOUHSC_01216	0.008987662	0.65	succinyl-CoA synthetase subunit beta
SAOUHSC_00953	0.002073981	0.64	diacylglycerol glucosyltransferase

SAOUHSC_01688	0.006321699	0.64	GTP-binding protein LepA
SAOUHSC_01262	0.015864429	0.64	recombinase A
SAOUHSC_01218	0.006474078	0.62	succinyl-CoA synthetase subunit alpha
SAOUHSC_01840	0.008262036	0.62	transglycosylase domain-containing protein
SAOUHSC_01322	0.012466264	0.62	homoserine kinase
SAOUHSC_01662	0.000265701	0.61	RNA polymerase sigma factor RpoD
SAOUHSC_02299	0.045510877	0.61	serine-protein kinase RsbW
SAOUHSC_02597	0.000797436	0.6	PTS system transporter
SAOUHSC_01774	0.005237840	0.6	porphobilinogen deaminase
SAOUHSC_02385	0.006165429	0.6	mannose-6-phosphate isomerase
SAOUHSC_01748	0.009121247	0.6	queuine tRNA-ribosyltransferase
SAOUHSC_01046	0.018116903	0.58	ABC transporter
SAOUHSC_01474	0.001054568	0.57	tRNA CCA-pyrophosphorylase
SAOUHSC_02337	0.009835897	0.57	UDP-N-acetylglucosamine 1-carboxyvinyltransferase
SAOUHSC_00908	0.000515082	0.56	coenzyme A disulfide reductase
SAOUHSC_02154	0.009530449	0.56	ABC transporter ATP-binding protein
SAOUHSC_00895	0.037279435	0.55	glutamate dehydrogenase
SAOUHSC_01473	0.008425376	0.54	bifunctional biotin operon

			repressor/biotin--[acetyl-CoA-carboxylase] synthetase BirA
SAOUHSC_01168	0.000817374	0.52	dihydroorotase
SAOUHSC_01502	0.030043662	0.51	ATP-dependent DNA helicase RecQ
SAOUHSC_02978	0.006100855	0.47	phage infection protein
SAOUHSC_01320	0.014487314	0.47	homoserine dehydrogenase
SAOUHSC_00641	0.023010872	0.46	teichoic acids export protein ATP-binding subunit
SAOUHSC_01041	0.023973606	0.46	pyruvate dehydrogenase complex. E1 component subunit beta
SAOUHSC_02314	0.048440522	0.45	sensor protein KdpD
SAOUHSC_00741	0.043600927	0.44	ribonucleotide reductase stimulatory protein
SAOUHSC_01811	0.013582697	0.42	DNA polymerase III subunit alpha superfamily protein
SAOUHSC_01962	0.019985828	0.42	uroporphyrinogen decarboxylase
SAOUHSC_01753	0.011470948	0.41	GTPase ObgE
SAOUHSC_00983	0.010455577	0.39	2-succinyl-6-hydroxy-2.4-cyclohexadiene-1-carboxylic acid synthase/2-oxoglutarate decarboxylase
SAOUHSC_00782	0.047965674	0.39	prolipoprotein diacylglyceryl transferase

SAOUHSC_02921	0.034888402	0.37	alpha-acetolactate decarboxylase
SAOUHSC_00905	0.044063123	-0.32	ATP-dependent nuclease subunit A
SAOUHSC_01147	0.027452162	-0.37	UDP-N-acetylmuramoyl-L-alanyl-D-glutamate synthetase
SAOUHSC_01856	0.045803620	-0.37	UDP-N-acetylmuramate--L-alanine ligase
SAOUHSC_02122	0.020009524	-0.4	NAD-dependent DNA ligase
SAOUHSC_00574	0.040489846	-0.41	phosphotransacetylase
SAOUHSC_01235	0.034357013	-0.43	uridylate kinase
SAOUHSC_01222	0.005491239	-0.46	DNA topoisomerase I
SAOUHSC_02084	0.030176481	-0.46	phage repressor protein
SAOUHSC_01737	0.037770330	-0.46	aspartyl-tRNA synthetase
SAOUHSC_00450	0.045455953	-0.46	Orn/Lys/Arg decarboxylase
SAOUHSC_02123	0.004125916	-0.47	ATP-dependent DNA helicase PcrA
SAOUHSC_02108	0.012954915	-0.47	ferritin
SAOUHSC_01199	0.030124034	-0.47	3-oxoacyl-(acyl-carrier-protein) reductase
SAOUHSC_01207	0.048337936	-0.48	signal recognition particle protein
SAOUHSC_02509	0.006808411	-0.49	50S ribosomal protein L2
SAOUHSC_00516	0.046884701	-0.49	preprotein translocase subunit SecE
SAOUHSC_02317	0.011970539	-0.5	UDP-N-acetylmuramoylalanyl-D-glutamyl-2.6-

			diaminopimelate--D-alanyl-D-alanyl ligase
SAOUHSC_00223	0.020359159	-0.51	teichoic acid biosynthesis protein F
SAOUHSC_02956	0.013835167	-0.52	nisin susceptibility-associated DNA-binding response regulator
SAOUHSC_00640	0.034888402	-0.52	teichoic acid biosynthesis protein
SAOUHSC_00724	0.017513410	-0.53	chorismate binding protein
SAOUHSC_01879	0.010168618	-0.54	virulence factor regulator protein
SAOUHSC_01741	0.037546490	-0.55	D-tyrosyl-tRNA(Tyr) deacylase
SAOUHSC_02577	0.041189388	-0.55	D-isomer specific 2-hydroxyacid dehydrogenase NAD binding domain-containing protein
SAOUHSC_01646	0.004035255	-0.56	glucokinase
SAOUHSC_03030	0.025832427	-0.56	sodium. sulfate symporter
SAOUHSC_00067	0.037629459	-0.56	L-lactate permease
SAOUHSC_00151	0.047943016	-0.56	branched-chain amino acid transport system II carrier protein
SAOUHSC_01403	0.000595211	-0.57	cold shock protein
SAOUHSC_02698	0.002459214	-0.57	amino acid ABC transporter permease
SAOUHSC_01933	0.006728148	-0.57	type I restriction-modification system subunit M

SAOUHSC_01481	0.000695562	-0.58	3-phosphoshikimate 1-carboxyvinyltransferase
SAOUHSC_02955	0.006388224	-0.58	nisin susceptibility-associated sensor histidine kinase
SAOUHSC_01980	0.007866510	-0.58	DNA-binding response regulator
SAOUHSC_01487	0.035816936	-0.58	ubiquinone/menaquinone biosynthesis methyltransferase
SAOUHSC_01624	0.008894939	-0.59	acetyl-CoA carboxylase biotin carboxyl carrier protein subunit
SAOUHSC_02318	0.010166560	-0.59	D-alanyl-alanine synthetase A
SAOUHSC_01234	0.028450376	-0.59	elongation factor Ts
SAOUHSC_01486	0.000750660	-0.61	heptaprenyl diphosphate syntase component II
SAOUHSC_01205	0.001204746	-0.61	signal recognition particle-docking protein FtsY
SAOUHSC_02740	0.001332617	-0.61	drug transporter
SAOUHSC_01225	0.008680633	-0.61	ATP-dependent protease peptidase subunit
SAOUHSC_01598	0.035062819	-0.61	AtsA/ElaC family protein
SAOUHSC_01766	0.000845481	-0.62	folylpolyglutamate synthase/dihydrofolate synthase
SAOUHSC_00988	0.011987129	-0.62	glutamyl endopeptidase
SAOUHSC_01593	0.041919149	-0.62	NUDIX domain-containing protein

SAOUHSC_01623	0.027218854	-0.63	acetyl-CoA carboxylase biotin carboxylase subunit
SAOUHSC_01143	0.011810114	-0.64	16S rRNA (cytosine(1402)-N(4))-methyltransferase
SAOUHSC_02484	0.000122799	-0.65	50S ribosomal protein L17
SAOUHSC_02491	0.002371107	-0.65	preprotein translocase subunit SecY
SAOUHSC_02943	0.011915775	-0.65	citrate transporter
SAOUHSC_02641	0.020818546	-0.65	permease domain-containing protein
SAOUHSC_00158	0.014820073	-0.66	PTS system transporter
SAOUHSC_01889	0.004931834	-0.67	riboflavin biosynthesis protein RibD
SAOUHSC_00562	0.037267055	-0.67	phosphomethylpyrimidine kinase
SAOUHSC_01107	0.013136237	-0.68	nucleoside-triphosphatase
SAOUHSC_00310	0.035062819	-0.68	PTS system ascorbate-specific transporter subunit IIC
SAOUHSC_02573	0.007111518	-0.69	Na+/H+ antiporter NhaC
SAOUHSC_01653	0.025672376	-0.69	superoxide dismutase
SAOUHSC_00752	0.000453759	-0.7	UDP-N-acetylenolpyruvoylglucosamine reductase
SAOUHSC_01620	0.013498391	-0.7	exodeoxyribonuclease VII large subunit
SAOUHSC_02687	0.022300474	-0.7	formate/nitrite transporter
SAOUHSC_01282	0.026037091	-0.7	glutathione peroxidase

SAOUHSC_01352	0.005021628	-0.71	DNA topoisomerase IV subunit A
SAOUHSC_02607	0.021022886	-0.71	urocanate hydratase
SAOUHSC_01493	0.000301205	-0.72	30S ribosomal protein S1
SAOUHSC_00186	0.001736452	-0.72	lipoprotein
SAOUHSC_02092	0.002897809	-0.72	aminopeptidase PepS
SAOUHSC_00538	0.010157839	-0.72	haloacid dehalogenase-like hydrolase
SAOUHSC_01316	0.024078553	-0.72	thermonuclease
SAOUHSC_00170	0.008425376	-0.73	RGD-containing lipoprotein
SAOUHSC_02549	0.040610681	-0.74	molybdenum ABC transporter substrate-binding protein
SAOUHSC_00956	0.024456727	-0.75	peptide chain release factor 3
SAOUHSC_01198	0.000852299	-0.77	malonyl CoA-acyl carrier protein transacylase
SAOUHSC_02942	0.002371297	-0.77	anaerobic ribonucleoside triphosphate reductase
SAOUHSC_01635	0.005651245	-0.77	shikimate kinase
SAOUHSC_02490	0.000195417	-0.78	adenylate kinase
SAOUHSC_01870	0.000268973	-0.78	16S rRNA pseudouridine(516) synthase
SAOUHSC_02947	0.000297546	-0.78	sulfite reductase (NADPH) flavoprotein subunit alpha
SAOUHSC_01095	0.000431799	-0.79	ribonuclease HIII

SAOUHSC_02276	0.000152759	-0.8	MutS domain-containing protein
SAOUHSC_00613	0.000810797	-0.8	iron compound ABC transporter substrate-binding protein
SAOUHSC_02133	0.000216650	-0.81	nicotinate phosphoribosyltransferase
SAOUHSC_01625	0.000537353	-0.81	elongation factor P
SAOUHSC_00526	0.000898749	-0.81	50S ribosomal protein L7Ae-like protein
SAOUHSC_02507	0.000125636	-0.83	50S ribosomal protein L22
SAOUHSC_00564	0.000487232	-0.83	uracil-DNA glycosylase
SAOUHSC_00517	0.008323574	-0.83	transcription antitermination protein
SAOUHSC_01773	0.000134294	-0.84	uroporphyrinogen III synthase
SAOUHSC_01329	0.000603279	-0.84	30S ribosomal protein S14
SAOUHSC_02132	0.007627639	-0.84	NAD synthetase
SAOUHSC_01280	0.000308650	-0.86	tRNA delta(2)-isopentenylpyrophosphate transferase
SAOUHSC_01626	0.001053299	-0.86	proline dipeptidase
SAOUHSC_02134	0.000368569	-0.88	nitric oxide synthase oxygenase subunit
SAOUHSC_02918	0.000628358	-0.88	pantoate--beta-alanine ligase
SAOUHSC_00799	0.000160673	-0.89	phosphopyruvate hydratase
SAOUHSC_00942	0.000842474	-0.89	GTP pyrophosphokinase
SAOUHSC_00921	0.000900982	-0.89	3-oxoacyl- synthase

SAOUHSC_02354	0.004453293	-0.89	serine hydroxymethyltransferase
SAOUHSC_01482	0.000433680	-0.9	3-dehydroquinate synthase
SAOUHSC_00536	0.002221829	-0.91	branched-chain amino acid aminotransferase
SAOUHSC_00798	0.026803916	-0.91	2.3-bisphosphoglycerate-independent phosphoglycerate mutase
SAOUHSC_01597	0.005350375	-0.92	pyrroline-5-carboxylate reductase
SAOUHSC_02958	0.011206893	-0.92	alkaline phosphatase III
SAOUHSC_01395	0.011314057	-0.92	aspartate semialdehyde dehydrogenase
SAOUHSC_02345	0.000748684	-0.93	F0F1 ATP synthase subunit alpha
SAOUHSC_00510	0.002448331	-0.93	serine acetyltransferase
SAOUHSC_00426	0.005062895	-0.94	ABC transporter substrate-binding protein
SAOUHSC_02972	0.000139815	-0.95	immunodominant antigen B
SAOUHSC_02127	0.002045615	-0.95	staphopain thiol proteinase
SAOUHSC_02380	0.002965909	-0.95	purine nucleoside phosphorylase
SAOUHSC_01226	0.000583575	-0.96	ATP-dependent protease ATP-binding subunit HslU
SAOUHSC_01496	0.000115294	-0.97	cytidylate kinase

SAOUHSC_00836	0.000571519	-0.97	glycine cleavage system protein H
SAOUHSC_01619	0.003760602	-0.97	exodeoxyribonuclease VII small subunit
SAOUHSC_02791	0.000515082	-0.98	pyrophosphohydrolase
SAOUHSC_00994	0.009827995	-0.98	bifunctional autolysin
SAOUHSC_01743	0.016612828	-0.98	adenine phosphoribosyltransferase
SAOUHSC_01435	0.000248907	-0.99	thymidylate synthase
SAOUHSC_02116	0.000334522	-0.99	aspartyl/glutamyl-tRNA amidotransferase subunit B
SAOUHSC_01392	0.000303666	-1.03	ABC transporter ATP-binding protein
SAOUHSC_01932	0.002210012	-1.03	type I restriction-modification system subunit S
SAOUHSC_03006	0.002210562	-1.08	lipase
SAOUHSC_02347	0.000173126	-1.09	F0F1 ATP synthase subunit B
SAOUHSC_00051	0.045561686	-1.1	1-phosphatidylinositol phosphodiesterase
SAOUHSC_01396	0.000625970	-1.11	4-hydroxy-tetrahydrodipicolinate synthase
SAOUHSC_01228	0.000198374	-1.12	transcriptional repressor CodY
SAOUHSC_00300	0.001461013	-1.12	lipase
SAOUHSC_01400	0.000474323	-1.13	alanine racemase
SAOUHSC_02021	0.009655343	-1.13	phi ETA orf 63-like protein
SAOUHSC_00157	0.000142308	-1.14	N-acetylmuramic acid-6-phosphate etherase

SAOUHSC_01274	0.000171986	-1.15	glycerol uptake operon antiterminator regulatory protein
SAOUHSC_01346	0.000218556	-1.15	glycine betaine transporter
SAOUHSC_02606	0.000342336	-1.17	imidazolonepropionase
SAOUHSC_01973	0.001055767	-1.17	3'-5' exoribonuclease YhaM
SAOUHSC_01177	0.000152230	-1.18	DNA-directed RNA polymerase subunit omega
SAOUHSC_00500	0.016622240	-1.2	glutamine amidotransferase subunit PdxT
SAOUHSC_02022	0.001018879	-1.26	phage tail fiber protein
SAOUHSC_00554	0.000171936	-1.28	6-phospho 3-hexuloisomerase
SAOUHSC_01112	0.002405560	-1.29	formyl peptide receptor-like 1 inhibitory protein
SAOUHSC_01397	0.000179353	-1.33	4-hydroxy-tetrahydrodipicolinate reductase
SAOUHSC_00671	0.002926150	-1.33	secretory antigen SsaA-like protein
SAOUHSC_01362	0.000888892	-1.36	4-oxalocrotonate tautomerase
SAOUHSC_01206	0.022971878	-1.41	DNA-binding protein
SAOUHSC_00424	0.000554950	-1.5	ABC transporter permease
SAOUHSC_02710	0.000163205	-1.51	leukocidin f subunit
SAOUHSC_01287	0.000187244	-1.55	glutamine synthetase
SAOUHSC_01549	0.009629544	-1.61	transcriptional activator rinB-like protein

SAOUHSC_02211	0.001991987	-1.62	phi PVL orf 50-like protein
SAOUHSC_02709	0.000101512	-1.78	leukocidin s subunit
SAOUHSC_00215	0.014037792	-1.88	PTS system transporter
SAOUHSC_01311	0.002426181	-1.94	ABC transporter ATP-binding protein
SAOUHSC_00229	0.000235169	-1.98	cell wall biosynthesis protein ScdA
SAOUHSC_02178	0.000437904	-2.93	phi PVL orf 22-like protein

Table S3. List of differentially expressed genes (DEG) uniquely identified in the 48h group. Genes were selected based on a false discovery rate (FDR) < 0.05 and an absolute $\log_2$ fold change ($|\log_2 FC|$) > 0.5. Only genes not differentially expressed in the 24h group under the same thresholds are included. Genes are sorted by their $\log_2$ fold change at 24 hours ("log2_Estimated_FoldChange").

Gene	FDR_adjusted_p_value	log2_Estimated_FoldChange	name
SAOUHSC_00132	0.02681716	2.31	aldehyde dehydrogenase
SAOUHSC_01012	0.036879081	2.17	phosphoribosylformylglycinamidine synthase I
SAOUHSC_01327	0.000190593	1.96	catalase
SAOUHSC_00195	0.000309499	1.82	acetyl-CoA acetyltransferase
SAOUHSC_01990	0.000106788	1.76	amino acid ABC transporter ATP-binding protein
SAOUHSC_02821	0.000868040	1.66	membrane spanning protein
SAOUHSC_01219	0.000475227	1.6	cell wall hydrolase
SAOUHSC_00627	0.029979769	1.49	monovalent cation/H+ antiporter subunit C
SAOUHSC_02012	0.000177261	1.39	glycosyltransferase

SAOUHSC_01008	0.001515314	1.39	5-(carboxyamino)imidazole ribonucleotide mutase
SAOUHSC_00898	0.007293705	1.37	argininosuccinate lyase
SAOUHSC_01708	0.005328035	1.28	LamB/YcsF family protein
SAOUHSC_00340	0.003161027	1.21	trans-sulfuration enzyme family protein
SAOUHSC_01710	0.002224978	1.17	acetyl-CoA carboxylase biotin carboxyl carrier protein subunit
SAOUHSC_00282	0.000832357	1.14	branched-chain amino acid transport system II carrier protein
SAOUHSC_02829	0.000109535	1.09	NAD(P)H-flavin oxidoreductase
SAOUHSC_02111	0.000145713	1.08	DNA polymerase IV
SAOUHSC_02238	0.024071693	1.08	phi PVL ORF 30-like protein
SAOUHSC_02461	0.010167259	1.07	MerR family transcriptional regulator
SAOUHSC_02809	0.000179381	1.03	gluconate operon transcriptional repressor
SAOUHSC_01941	0.021320061	1.03	serine protease SplB
SAOUHSC_02664	0.009708185	1.02	transcriptional regulator
SAOUHSC_00339	0.006491398	1	bifunctional homocysteine S-methyltransferase/5,10-methylenetetrahydrofolate reductase
SAOUHSC_01394	0.000549070	0.96	aspartate kinase
SAOUHSC_01009	0.000197411	0.94	5-(carboxyamino)imidazole ribonucleotide synthase
SAOUHSC_02219	0.010399688	0.92	phi ETA orf 20-like protein

SAOUHSC_01709	0.000955078	0.91	acetyl-CoA carboxylase biotin carboxylase
SAOUHSC_02170	0.001130164	0.91	peptidoglycan hydrolase
SAOUHSC_01997	0.007451031	0.91	ferric uptake regulator-like protein
SAOUHSC_02284	0.008997482	0.9	ketol-acid reductoisomerase
SAOUHSC_02808	0.007293705	0.89	gluconate kinase
SAOUHSC_01406	0.014799911	0.87	acylphosphatase
SAOUHSC_01013	0.003057620	0.86	phosphoribosylformylglycinamidine synthase II
SAOUHSC_01801	0.005657889	0.86	isocitrate dehydrogenase
SAOUHSC_02282	0.003538868	0.85	acetolactate synthase large subunit
SAOUHSC_01663	0.000246502	0.84	DNA primase
SAOUHSC_02285	0.002376868	0.84	2-isopropylmalate synthase
SAOUHSC_00466	0.004264390	0.84	4-diphosphocytidyl-2C-methyl-D-erythritol kinase
SAOUHSC_01833	0.005633525	0.83	D-3-phosphoglycerate dehydrogenase
SAOUHSC_01849	0.011205784	0.83	acetoin utilization protein AcuC
SAOUHSC_01281	0.001380854	0.82	host factor 1 protein
SAOUHSC_02483	0.002523915	0.82	cobalt transporter ATP-binding subunit
SAOUHSC_01901	0.001047062	0.81	putative translaldolase
SAOUHSC_02409	0.018962993	0.78	arginase
SAOUHSC_01099	0.001997181	0.77	recombination and DNA strand exchange inhibitor protein
SAOUHSC_02119	0.009847008	0.77	high affinity proline permease

SAOUHSC_00020	0.002338824	0.74	two-component response regulator
SAOUHSC_02287	0.018662047	0.74	isopropylmalate isomerase large subunit
SAOUHSC_02137	0.004103200	0.71	sodium-dependent transporter
SAOUHSC_01606	0.008624415	0.71	peptidase T
SAOUHSC_00729	0.002126921	0.7	ABC transporter ATP-binding protein
SAOUHSC_01685	0.002613322	0.69	heat-inducible transcription repressor HrcA
SAOUHSC_01686	0.018280911	0.69	coproporphyrinogen III oxidase
SAOUHSC_00652	0.001533321	0.67	iron compound ABC transporter ATP-binding protein
SAOUHSC_00001	0.005895051	0.65	chromosomal replication initiation protein
SAOUHSC_00653	0.016280642	0.65	ferrichrome transport permease FhuB
SAOUHSC_02216	0.027532805	0.65	phage DnaC-like protein
SAOUHSC_00628	0.013922333	0.64	monovalent cation/H+ antiporter subunit D
SAOUHSC_02924	0.037547852	0.64	4-aminobutyrate aminotransferase
SAOUHSC_02003	0.024209602	0.63	ABC transporter ATP-binding/permease
SAOUHSC_02286	0.034080141	0.63	3-isopropylmalate dehydrogenase
SAOUHSC_01615	0.018962993	0.62	DNA repair protein RecN
SAOUHSC_01432	0.039394592	0.62	methionine sulfoxide reductase A

SAOUHSC_00927	0.017859595	0.6	oligopeptide ABC transporter substrate-binding protein
SAOUHSC_02389	0.011604962	0.57	cation efflux family protein
SAOUHSC_00222	0.006531735	0.56	teichoic acid biosynthesis protein TagB
SAOUHSC_00743	0.049590119	0.53	ribonucleotide-diphosphate reductase subunit beta
SAOUHSC_00220	0.036757098	0.48	2-C-methyl-D-erythritol 4-phosphate cytidylyltransferase
SAOUHSC_00769	0.036879080	0.45	preprotein translocase subunit SecA
SAOUHSC_02405	0.049590119	-0.39	phosphoglucosamine mutase
SAOUHSC_01053	0.038982388	-0.41	manganese transport protein MntH
SAOUHSC_02926	0.031745831	-0.44	fructose-1,6-bisphosphate aldolase
SAOUHSC_01779	0.025132033	-0.45	trigger factor
SAOUHSC_01434	0.049590119	-0.45	dihydrofolate reductase
SAOUHSC_01633	0.038329563	-0.46	glycine dehydrogenase subunit 1
SAOUHSC_01002	0.049563768	-0.47	quinol oxidase AA3 subunit II
SAOUHSC_01820	0.022562633	-0.48	acetate kinase
SAOUHSC_00524	0.023037571	-0.5	DNA-directed RNA polymerase subunit beta
SAOUHSC_02916	0.032700328	-0.51	aspartate alpha-decarboxylase
SAOUHSC_02375	0.048460698	-0.53	S-ribosylhomocysteinase
SAOUHSC_02976	0.036290643	-0.55	mannose-6-phosphate isomerase

SAOUHSC_02343	0.011604962	-0.56	F0F1 ATP synthase subunit gamma
SAOUHSC_01093	0.036510521	-0.56	phenylalanyl-tRNA synthetase subunit beta
SAOUHSC_03051	0.007010430	-0.57	16S rRNA methyltransferase GidB
SAOUHSC_02400	0.041042213	-0.57	PTS system mannitol-specific protein
SAOUHSC_00866	0.010622331	-0.58	D-isomer specific 2-hydroxyacid dehydrogenase NAD binding domain-containing protein
SAOUHSC_02371	0.014797503	-0.59	pantothenate kinase
SAOUHSC_02582	0.011604962	-0.61	formate dehydrogenase subunit alpha
SAOUHSC_02336	0.026042648	-0.61	(3R)-hydroxymyristoyl-ACP dehydratase
SAOUHSC_02801	0.029979769	-0.61	UTP-glucose-1-phosphate uridylyltransferase
SAOUHSC_02962	0.038260081	-0.61	tributyrin esterase
SAOUHSC_00556	0.004581520	-0.62	proline/betaine transporter
SAOUHSC_01114	0.038467795	-0.62	fibrinogen-binding protein
SAOUHSC_00204	0.007499319	-0.63	globin domain-containing protein
SAOUHSC_02742	0.044882791	-0.64	amino acid transporter
SAOUHSC_02361	0.003395960	-0.65	50S ribosomal protein L31 type B
SAOUHSC_00128	0.011420695	-0.65	cap5O protein/UDP-N-acetyl-D-mannosaminuronic acid dehydrogenase

SAOUHSC_01827	0.017965834	-0.66	septation ring formation regulator EzrA
SAOUHSC_01632	0.000483867	-0.67	glycine dehydrogenase subunit 2
SAOUHSC_00579	0.003380518	-0.67	phosphomevalonate kinase
SAOUHSC_02366	0.011375123	-0.67	fructose-bisphosphate aldolase
SAOUHSC_01171	0.016625780	-0.68	orotidine 5'-phosphate decarboxylase
SAOUHSC_02512a	0.018962993	-0.68	30S ribosomal protein S10
SAOUHSC_00520	0.007010430	-0.69	50S ribosomal protein L10
SAOUHSC_01678	0.010737024	-0.69	30S ribosomal protein S21
SAOUHSC_01211	0.041031143	-0.69	50S ribosomal protein L19
SAOUHSC_01191	0.004420540	-0.7	50S ribosomal protein L28
SAOUHSC_01815	0.009001677	-0.7	metal-dependent hydrolase
SAOUHSC_02967	0.027552683	-0.7	arginine/ornithine antiporter
SAOUHSC_02363	0.007492706	-0.71	aldehyde dehydrogenase
SAOUHSC_02629	0.000414551	-0.73	EmrB/QacA family drug resistance transporter
SAOUHSC_01651	0.004347754	-0.73	50S ribosomal protein L33
SAOUHSC_01586	0.003197171	-0.74	DNA-binding response regulator
SAOUHSC_00019	0.007451031	-0.74	adenylosuccinate synthetase
SAOUHSC_00719	0.013626865	-0.74	7-cyano-7-deazaguanosine (preQ0)

			biosynthesis protein QueE
SAOUHSC_02648	0.004548428	-0.76	L-lactate permease
SAOUHSC_02565	0.002687850	-0.77	urease accessory protein UreD
SAOUHSC_00469	0.007481948	-0.8	regulatory protein SpoVG
SAOUHSC_02403	0.000607840	-0.81	mannitol-1-phosphate 5-dehydrogenase
SAOUHSC_02340	0.003037208	-0.81	F0F1 ATP synthase subunit epsilon
SAOUHSC_01164	0.009364114	-0.81	bifunctional pyrimidine regulatory protein PyrR/uracil phosphoribosyltransferase
SAOUHSC_00422	0.003451971	-0.82	trans-sulfuration enzyme family protein
SAOUHSC_02265	0.018762023	-0.82	accessory gene regulator protein A
SAOUHSC_02333	0.024874900	-0.83	transglycosylase SceD
SAOUHSC_01490	0.004980935	-0.84	DNA-binding protein HU
SAOUHSC_02559	0.006165918	-0.84	urease subunit beta
SAOUHSC_00720	0.014220087	-0.84	6-pyruvoyl tetrahydropterin synthase
SAOUHSC_02402	0.003865271	-0.85	PTS system mannitol-specific transporter subunit IIA
SAOUHSC_02849	0.001515314	-0.86	pyruvate oxidase
SAOUHSC_00216	0.044386517	-0.87	PTS system transporter
SAOUHSC_01065	0.000671432	-0.88	heme A synthase
SAOUHSC_02489	0.000796392	-0.88	translation initiation factor IF-1

SAOUHSC_00634	0.026632818	-0.88	ABC transporter substrate-binding protein
SAOUHSC_T00029	0.003685758	-0.9	tRNA-Ile
SAOUHSC_00796	0.017859940	-0.9	phosphoglycerate kinase
SAOUHSC_02244	0.002434526	-0.91	succinyl-diaminopimelate desuccinylase
SAOUHSC_01121	0.032999495	-0.92	alpha-hemolysin
SAOUHSC_01110	0.000112964	-0.94	fibrinogen-binding protein-like protein
SAOUHSC_02444	0.000905906	-0.94	BCCT family osmoprotectant transporter
SAOUHSC_02562	0.000147020	-0.96	urease accessory protein UreE
SAOUHSC_02708	0.000409379	-0.97	gamma-hemolysin h-gamma-II subunit
SAOUHSC_02512	0.000591142	-0.98	50S ribosomal protein L3
SAOUHSC_00183	0.009369362	-1	sugar phosphate antiporter
SAOUHSC_00528	0.018962993	-1	30S ribosomal protein S7
SAOUHSC_00350	0.018701027	-1.01	30S ribosomal protein S18
SAOUHSC_01960	0.022562633	-1.01	protoporphyrinogen oxidase
SAOUHSC_02571	0.001173580	-1.02	secretory antigen
SAOUHSC_02968	0.005744153	-1.07	ornithine carbamoyltransferase
SAOUHSC_01279	0.042837022	-1.07	hydrolase alpha/beta fold domain-containing protein
SAOUHSC_02503	0.002189951	-1.09	30S ribosomal protein S17

SAOUHSC_02965	0.001190512	-1.1	carbamate kinase
SAOUHSC_T00028	0.002238138	-1.11	tRNA-Ile
SAOUHSC_02061	0.008997482	-1.12	phi PVL orf 50-like protein
SAOUHSC_00533	0.000297635	-1.17	chaperone protein HchA
SAOUHSC_01385	0.000719943	-1.17	phosphate transporter ATP-binding protein
SAOUHSC_00122	0.000388215	-1.37	capsular polysaccharide biosynthesis protein Cap5I
SAOUHSC_01278	0.000212464	-1.43	aerobic glycerol-3-phosphate dehydrogenase
SAOUHSC_01542	0.006524082	-1.43	SNF2 family protein
SAOUHSC_01389	0.000406978	-1.56	phosphate ABC transporter substrate-binding protein
SAOUHSC_01571	0.000207961	-1.64	SLT orf 71-like protein
SAOUHSC_01543	0.004762622	-1.68	phi-like protein
SAOUHSC_01563	0.000712517	-1.73	phage encoded DNA polymerase I
SAOUHSC_01524	0.002256919	-1.76	holin-like protein
SAOUHSC_01523	0.003067580	-1.81	SLT orf 527-like protein
SAOUHSC_01539	0.011205784	-1.83	terminase small subunit
SAOUHSC_01570	0.000306708	-1.87	PVL orf 37-like protein
SAOUHSC_01538	0.001015172	-1.88	phage terminase large subunit
SAOUHSC_01386	0.001324073	-1.99	phosphate ABC transporter permease
SAOUHSC_01525	0.000109535	-2.01	phage tail tape meausure protein
SAOUHSC_01521	0.038669542	-2.07	SLT orf 636-like protein

SAOUHSC_01537	0.000446570	-2.08	HK97 family phage portal protein
SAOUHSC_01532	0.003732061	-2.19	SLT orf 110-like protein
SAOUHSC_01536	0.000101284	-2.33	scaffolding protease
SAOUHSC_01516	0.000287073	-2.41	holin protein
SAOUHSC_01515	0.008840927	-2.57	petidoglycan hydrolase

Table S 4. List of differentially expressed genes (DEG) identified in DESeq2 analysis between both exposed groups (24h vs. 48h). Genes were selected based on a false discovery rate (FDR) < 0.05 and an absolute $\log_2$ fold change ($|\log_2 FC|$) > 0.5. Genes are sorted by their $\log_2$ fold change ("log2_Estimated_FoldChange").

Gene	FDR_adjusted_p_value	log2_Estimated_Fold Change	name
SAOUHSC_02260	7.55E-78	5.84	delta-hemolysin
SAOUHSC_02181	5.14E-10	3.53	phi PVL orfs 18-19-like protein
SAOUHSC_00206	6.00E-26	3.51	L-lactate dehydrogenase
SAOUHSC_02171	0.004538197	3.26	staphylokinase
SAOUHSC_01531	6.833E-08	3.19	SLT orf 123-like protein
SAOUHSC_00533	2.11E-31	3.1	chaperone protein HchA
SAOUHSC_01452	1.715E-08	3.1	alanine dehydrogenase
SAOUHSC_02178	0.013168866	3.05	phi PVL orf 22-like protein
SAOUHSC_01515	0.000004047	2.89	peptidoglycan hydrolase
SAOUHSC_02182	8.633E-09	2.87	tail length tape measure protein
SAOUHSC_00708	1.17E-18	2.83	fructose specific permease
SAOUHSC_01536	5.40E-06	2.79	scaffolding protease
SAOUHSC_01788	2.04E-18	2.78	threonyl-tRNA synthetase
SAOUHSC_01529	0.000013669	2.64	major tail protein
SAOUHSC_01532	0.002171498	2.61	SLT orf 110-like protein
SAOUHSC_00707	6.64E-10	2.6	1-phosphofructokinase
SAOUHSC_01278	4.23E-24	2.58	aerobic glycerol-3-phosphate dehydrogenase
SAOUHSC_00608	4.13E-14	2.55	alcohol dehydrogenase
SAOUHSC_01516	0.003649629	2.53	holin protein
SAOUHSC_00199	1.527E-06	2.51	acyl CoA:acetate/3-ketoacid CoA transferase
SAOUHSC_01519	0.001780222	2.48	SLT orf 129-like protein
SAOUHSC_01451	0.000114799	2.46	threonine dehydratase

SAOUHSC_01528	0.000102403	2.44	bacteriophage L54aIg-like domain-containing protein
SAOUHSC_01521	0.000060932	2.41	SLT orf 636-like protein
SAOUHSC_01525	4.42E-06	2.4	phage tail tape meausure protein
SAOUHSC_01538	0.000107874	2.37	phage terminase large subunit
SAOUHSC_02173	1.42E-06	2.34	amidase
SAOUHSC_01520	0.000330827	2.33	SLT orf 488-like protein
SAOUHSC_00899	0.000252616	2.22	argininosuccinate synthase
SAOUHSC_02648	1.59E-17	2.17	L-lactate permease
SAOUHSC_02180	0.000011523	2.16	phage minor structural protein
SAOUHSC_00195	0.000152741	2.14	acetyl-CoA acetyltransferase
SAOUHSC_00898	0.000462807	2.14	argininosuccinate lyase
SAOUHSC_01666	5.08E-09	2.1	glycyl-tRNA synthetase
SAOUHSC_01539	0.028758370	2.07	terminase small subunit
SAOUHSC_00927	9.86E-09	2.03	oligopeptide ABC transporter substrate-binding protein
SAOUHSC_01990	0.000746072	2.03	amino acid ABC transporter ATP-binding protein
SAOUHSC_02174	0.001610794	2.03	phage phi LC3 family holin
SAOUHSC_01524	0.000326190	2.01	holin-like protein
SAOUHSC_01114	1.71E-07	1.96	fibrinogen-binding protein
SAOUHSC_01394	7.15E-11	1.93	aspartate kinase
SAOUHSC_00926	6.73E-06	1.9	oligopeptide ABC transporter ATP-binding protein
SAOUHSC_01537	0.002312151	1.88	HK97 family phage portal protein
SAOUHSC_00215	0.006275525	1.88	PTS system transporter
SAOUHSC_02468	8.126E-13	1.87	acetolactate synthase
SAOUHSC_01395	1.36E-06	1.87	aspartate semialdehyde dehydrogenase
SAOUHSC_02265	2.448E-07	1.85	accessory gene regulator protein A
SAOUHSC_01396	4.806E-08	1.84	4-hydroxy-tetrahydrodipicolinate synthase
SAOUHSC_01397	0.000028263	1.84	4-hydroxy-tetrahydrodipicolinate reductase
SAOUHSC_00871	0.005161639	1.84	D-alanine--poly(phosphoribitol) ligase subunit 2
SAOUHSC_01523	0.003443846	1.83	SLT orf 527-like protein
SAOUHSC_02467	2.371E-11	1.82	alpha-acetolactate decarboxylase
SAOUHSC_02282	3.71E-10	1.8	acetolactate synthase large subunit
SAOUHSC_02333	7.439E-07	1.8	transglycosylase SceD
SAOUHSC_00933	6.10E-06	1.8	tryptophanyl-tRNA synthetase
SAOUHSC_01889	0.003816830	1.8	riboflavin biosynthesis protein RibD
SAOUHSC_02264	5.08E-07	1.79	accessory gene regulator protein C
SAOUHSC_01012	0.014880709	1.72	phosphoribosylformylglycinamidine synthase I
SAOUHSC_01285	6.15E-07	1.71	glutamine synthetase repressor
SAOUHSC_02830	1.42E-09	1.7	D-lactate dehydrogenase

SAOUHSC_02285	9.79E-09	1.7	2-isopropylmalate synthase
SAOUHSC_02284	3.96E-06	1.7	ketol-acid reductoisomerase
SAOUHSC_01327	0.002808540	1.7	catalase
SAOUHSC_00818	0.019288114	1.69	thermonuclease
SAOUHSC_02281	5.80E-07	1.68	dihydroxy-acid dehydratase
SAOUHSC_01991	0.002980443	1.65	ABC transporter permease
SAOUHSC_02283	0.008577146	1.65	acetolactate synthase 1 regulatory subunit
SAOUHSC_00861	0.014415076	1.65	lipoyl synthase
SAOUHSC_02887	0.002952575	1.64	immunodominant antigen A
SAOUHSC_01398	6.15E-07	1.62	2,3,4,5-tetrahydropyridine-2,6-dicarboxylate N-acetyltransferase
SAOUHSC_02287	2.526E-07	1.6	isopropylmalate isomerase large subunit
SAOUHSC_01708	0.000866629	1.58	LamB/YcsF family protein
SAOUHSC_01347	0.002952575	1.58	aconitate hydratase
SAOUHSC_02286	4.509E-07	1.57	3-isopropylmalate dehydrogenase
SAOUHSC_02288	0.000534354	1.5	3-isopropylmalate dehydratase small subunit
SAOUHSC_01287	0.000930181	1.5	glutamine synthetase
SAOUHSC_00009	0.002732215	1.5	seryl-tRNA synthetase
SAOUHSC_00231	0.000717844	1.49	two-component response regulator
SAOUHSC_00544	1.035E-06	1.46	fibrinogen-binding protein SdrC
SAOUHSC_02211	0.022457146	1.46	phi PVL orf 50-like protein
SAOUHSC_01093	1.23E-06	1.45	phenylalanyl-tRNA synthetase subunit beta
SAOUHSC_02862	0.001145235	1.45	ATP-dependent Clp protease ATP-binding subunit ClpC
SAOUHSC_00671	0.003251256	1.44	secretory antigen SsaA-like protein
SAOUHSC_00921	3.98E-06	1.41	3-oxoacyl- synthase
SAOUHSC_00115	3.29E-07	1.4	capsular polysaccharide biosynthesis protein Cap5B
SAOUHSC_02022	0.003623869	1.4	phage tail fiber protein
SAOUHSC_01092	6.61E-07	1.39	phenylalanyl-tRNA synthetase subunit alpha
SAOUHSC_02576	0.001871187	1.39	secretory antigen SsaA
SAOUHSC_02244	0.000024730	1.35	succinyl-diaminopimelate desuccinylase
SAOUHSC_02968	0.000619553	1.33	ornithine carbamoyltransferase
SAOUHSC_00284	0.002557390	1.33	5'-nucleotidase
SAOUHSC_01159	0.000021306	1.32	isoleucyl-tRNA synthetase
SAOUHSC_02969	0.000107921	1.3	arginine deiminase
SAOUHSC_00214	0.032107481	1.29	PTS system transporter subunit IIA
SAOUHSC_03006	0.000142218	1.28	lipase
SAOUHSC_01887	0.000224930	1.28	riboflavin biosynthesis protein
SAOUHSC_01362	0.012452550	1.26	4-oxalocrotonate tautomerase
SAOUHSC_01875	4.69E-07	1.25	leucyl-tRNA synthetase
SAOUHSC_01401	0.000049869	1.25	diaminopimelate decarboxylase

SAOUHSC_00513	0.000743914	1.24	23S rRNA (guanosine(2251)-2'-O)-methyltransferase RlmB
SAOUHSC_01276	3.732E-06	1.23	glycerol kinase
SAOUHSC_01888	1.16E-07	1.21	riboflavin synthase subunit alpha
SAOUHSC_01225	0.000558206	1.21	ATP-dependent protease peptidase subunit
SAOUHSC_01223	0.000787785	1.21	tRNA (uracil-5-)-methyltransferase Gid
SAOUHSC_02965	0.000179884	1.2	carbamate kinase
SAOUHSC_01272	0.000021306	1.18	DNA mismatch repair protein MutS
SAOUHSC_01282	0.000930616	1.17	glutathione peroxidase
SAOUHSC_01651	0.000218032	1.16	50S ribosomal protein L33
SAOUHSC_02773	0.000651434	1.16	transporter
SAOUHSC_00511	0.000751384	1.16	cysteinyl-tRNA synthetase
SAOUHSC_01328	0.004560965	1.16	50S ribosomal protein L33
SAOUHSC_01580	0.013168866	1.16	phi PVL ORF 30-like protein
SAOUHSC_00536	0.000195872	1.14	branched-chain amino acid aminotransferase
SAOUHSC_01868	0.000017198	1.13	dipeptidase PepV
SAOUHSC_01585	0.000174647	1.13	respiratory response protein SrrB
SAOUHSC_01226	0.001517072	1.12	ATP-dependent protease ATP-binding subunit HslU
SAOUHSC_01400	0.006099924	1.12	alanine racemase
SAOUHSC_02706	0.036649825	1.12	immunoglobulin G-binding protein Sbi
SAOUHSC_00216	0.007200332	1.11	PTS system transporter
SAOUHSC_01389	0.046078498	1.11	phosphate ABC transporter substrate-binding protein
SAOUHSC_01191	0.000031931	1.1	50S ribosomal protein L28
SAOUHSC_01028	0.036500200	1.1	phosphocarrier protein HPr
SAOUHSC_01273	0.000522562	1.08	DNA mismatch repair protein
SAOUHSC_01678	0.001801753	1.08	30S ribosomal protein S21
SAOUHSC_00994	0.009799360	1.08	bifunctional autolysin
SAOUHSC_02629	0.000934010	1.07	EmrB/QacA family drug resistance transporter
SAOUHSC_00937	0.001065216	1.07	oligoendopeptidase F
SAOUHSC_01983	0.016370141	1.07	fumarate hydratase
SAOUHSC_01385	0.018428231	1.07	phosphate transporter ATP-binding protein
SAOUHSC_02920	0.000253966	1.06	2-dehydropantoate 2-reductase
SAOUHSC_01626	0.000988006	1.06	proline dipeptidase
SAOUHSC_01269	0.002189118	1.06	(dimethylallyl)adenosine tRNA methylthiotransferase
SAOUHSC_00116	0.006218109	1.06	capsular polysaccharide biosynthesis protein Cap8C
SAOUHSC_02729	0.021937951	1.06	amino acid ABC transporter-like protein
SAOUHSC_00120	0.000143513	1.05	UDP-N-acetylglucosamine 2-epimerase

SAOUHSC_02967	0.001378570	1.05	arginine/ornithine antiporter
SAOUHSC_01490	3.799E-07	1.04	DNA-binding protein HU
SAOUHSC_01497	0.000622540	1.04	L-asparaginase
SAOUHSC_00510	0.003650309	1.04	serine acetyltransferase
SAOUHSC_00869	0.001218025	1.03	D-alanine--poly(phosphoribitol) ligase subunit 1
SAOUHSC_01839	0.002026073	1.03	tyrosyl-tRNA synthetase
SAOUHSC_00117	0.000406316	1.02	capsular polysaccharide biosynthesis protein Cap5D
SAOUHSC_00187	0.034781657	1.02	formate acetyltransferase
SAOUHSC_01501	0.002138988	1.01	elastin binding protein
SAOUHSC_00836	0.001571509	1	glycine cleavage system protein H
SAOUHSC_02571	0.024811704	1	secretory antigen
SAOUHSC_01329	0.001072287	0.98	30S ribosomal protein S14
SAOUHSC_01071	0.001488534	0.98	glycerophosphoryl diester phosphodiesterase
SAOUHSC_01709	0.002452796	0.98	acetyl-CoA carboxylase biotin carboxylase
SAOUHSC_00217	0.012123841	0.98	sorbitol dehydrogenase
SAOUHSC_00872	0.001218025	0.97	extramembranal protein
SAOUHSC_00126	0.002811699	0.97	capsular polysaccharide biosynthesis protein Cap8M
SAOUHSC_00061	0.018796484	0.96	myosin-cross-reactive antigen
SAOUHSC_01886	0.037716012	0.96	6,7-dimethyl-8-ribityllumazine synthase
SAOUHSC_01107	0.002036017	0.94	nucleoside-triphosphatase
SAOUHSC_02405	0.001531578	0.93	phosphoglucosamine mutase
SAOUHSC_01867	0.002962431	0.93	D-alanine aminotransferase
SAOUHSC_01251	0.022266602	0.93	polynucleotide phosphorylase/polyadenylase
SAOUHSC_01471	0.001780222	0.92	asparaginyl-tRNA synthetase
SAOUHSC_02972	0.001401318	0.91	immunodominant antigen B
SAOUHSC_01586	0.003278541	0.91	DNA-binding response regulator
SAOUHSC_00521	0.017402751	0.91	50S ribosomal protein L7/L12
SAOUHSC_01310	0.044038731	0.91	cardiolipin synthetase
SAOUHSC_01386	0.009924681	0.9	phosphate ABC transporter permease
SAOUHSC_01597	0.016844314	0.9	pyrroline-5-carboxylate reductase
SAOUHSC_00114	0.042726572	0.9	capsular polysaccharide biosynthesis protein
SAOUHSC_02343	0.018428231	0.89	F0F1 ATP synthase subunit gamma
SAOUHSC_00520	0.010847771	0.88	50S ribosomal protein L10
SAOUHSC_02352	0.018097778	0.88	UDP-GlcNAc 2-epimerase
SAOUHSC_01228	0.019912677	0.88	transcriptional repressor CodY
SAOUHSC_02142	0.040133383	0.88	aldehyde dehydrogenase
SAOUHSC_00230	0.011407748	0.87	two-component sensor histidine kinase

SAOUHSC_02132	0.014672841	0.87	NAD synthetase
SAOUHSC_01177	0.022804637	0.87	DNA-directed RNA polymerase subunit omega
SAOUHSC_00118	0.026274147	0.87	capsular polysaccharide biosynthesis protein Cap5E
SAOUHSC_00119	0.006551111	0.86	capsular polysaccharide biosynthesis protein Cap8F
SAOUHSC_00020	0.000958548	0.85	two-component response regulator
SAOUHSC_02918	0.002690133	0.85	pantoate--beta-alanine ligase
SAOUHSC_02353	0.026490588	0.85	uracil phosphoribosyltransferase
SAOUHSC_00958	0.001384452	0.84	serine protease HtrA
SAOUHSC_00021	0.001540523	0.84	sensory box histidine kinase VicK
SAOUHSC_01280	0.013225965	0.84	tRNA delta(2)-isopentenylpyrophosphate transferase
SAOUHSC_00373	0.002585509	0.83	xanthine permease
SAOUHSC_00893	0.001531578	0.82	FMN oxidoreductase
SAOUHSC_01482	0.005282476	0.82	3-dehydroquinate synthase
SAOUHSC_00422	0.020039775	0.82	trans-sulfuration enzyme family protein
SAOUHSC_02380	0.031182813	0.82	purine nucleoside phosphorylase
SAOUHSC_01785	0.005117037	0.81	50S ribosomal protein L35
SAOUHSC_01224	0.010243926	0.81	site-specific recombinase
SAOUHSC_00799	0.014414606	0.81	phosphopyruvate hydratase
SAOUHSC_02375	0.018097778	0.81	S-ribosylhomocysteinase
SAOUHSC_02274	0.001969857	0.8	ABC transporter ATP-binding protein
SAOUHSC_00375	0.004421266	0.8	GMP synthase
SAOUHSC_02740	0.006372588	0.8	drug transporter
SAOUHSC_00017	0.024484450	0.8	50S ribosomal protein L9
SAOUHSC_01635	0.025305124	0.79	shikimate kinase
SAOUHSC_01346	0.035755292	0.79	glycine betaine transporter
SAOUHSC_01493	0.000090731	0.78	30S ribosomal protein S1
SAOUHSC_02962	0.002504254	0.78	tributyrin esterase
SAOUHSC_01198	0.004791060	0.78	malonyl CoA-acyl carrier protein transacylase
SAOUHSC_00956	0.043307762	0.78	peptide chain release factor 3
SAOUHSC_02347	0.021799455	0.77	F0F1 ATP synthase subunit B
SAOUHSC_01432	0.031642195	0.77	methionine sulfoxide reductase A
SAOUHSC_01149	0.000859499	0.76	cell division protein
SAOUHSC_01496	0.008681627	0.75	cytidylate kinase
SAOUHSC_01492	0.009073024	0.74	GTP-binding protein EngA
SAOUHSC_00613	0.013769606	0.74	iron compound ABC transporter substrate-binding protein
SAOUHSC_00342	0.036315382	0.73	ParB family chromosome partioning protein
SAOUHSC_01434	0.021799455	0.72	dihydrofolate reductase
SAOUHSC_02341	0.044954716	0.72	F0F1 ATP synthase subunit beta

SAOUHSC_00912	0.021455711	0.71	ATP-dependent Clp protease ATP-binding subunit ClpB
SAOUHSC_00155	0.033844096	0.71	PTS system glucose-specific protein
SAOUHSC_01267	0.009606774	0.7	2-oxoglutarate ferredoxin oxidoreductase subunit beta
SAOUHSC_02979	0.031394073	0.7	N-acetylmuramoyl-L-alanine amidase
SAOUHSC_00942	0.038280056	0.7	GTP pyrophosphokinase
SAOUHSC_02527	0.002370893	0.69	peptidoglycan pentaglycine interpeptide biosynthesis protein FmhB
SAOUHSC_01197	0.044778111	0.69	glycerol-3-phosphate acyltransferase PlsX
SAOUHSC_02318	0.003035886	0.68	D-alanyl-alanine synthetase A
SAOUHSC_02133	0.010352909	0.68	nicotinate phosphoribosyltransferase
SAOUHSC_01657	0.022044828	0.68	ABC transporter
SAOUHSC_01053	0.014879560	0.67	manganese transport protein MntH
SAOUHSC_01665	0.019573922	0.67	CBS domain-containing protein
SAOUHSC_02091	0.031394073	0.67	acyl-CoA thioesterase
SAOUHSC_01483	0.021203919	0.66	chorismate synthase
SAOUHSC_01364	0.031394073	0.66	prephenate dehydrogenase
SAOUHSC_03030	0.023778803	0.65	sodium, sulfate symporter
SAOUHSC_02403	0.042726572	0.65	mannitol-1-phosphate 5-dehydrogenase
SAOUHSC_00346	0.007686790	0.64	GTP-dependent nucleic acid-binding protein EngD
SAOUHSC_01771	0.043307762	0.64	glutamate-1-semialdehyde aminotransferase
SAOUHSC_00320	0.012546768	0.63	NADPH-dependent FMN reductase
SAOUHSC_00125	0.020745984	0.63	cap5L protein/glycosyltransferase
SAOUHSC_01981	0.023909467	0.63	sensor histidine kinase
SAOUHSC_01827	0.033406659	0.63	septation ring formation regulator EzrA
SAOUHSC_01481	0.005292567	0.62	3-phosphoshikimate 1-carboxyvinyltransferase
SAOUHSC_02123	0.015229325	0.62	ATP-dependent DNA helicase PcrA
SAOUHSC_02126	0.017316171	0.59	adenylosuccinate lyase
SAOUHSC_01646	0.036092782	0.59	glucokinase
SAOUHSC_02955	0.043931475	0.59	nisin susceptibility-associated sensor histidine kinase
SAOUHSC_00870	0.010338355	0.57	D-alanine transfer protein DltB
SAOUHSC_02916	0.030117892	0.57	aspartate alpha-decarboxylase
SAOUHSC_02456	0.033752189	0.56	lactose phosphotransferase system repressor
SAOUHSC_01201	0.043307762	0.55	acyl carrier protein
SAOUHSC_01420	0.027389509	0.54	DNA-binding response regulator

SAOUHSC_00374	0.030619335	0.54	inosine-5'-monophosphate dehydrogenase
SAOUHSC_01222	0.033735908	0.54	DNA topoisomerase I
SAOUHSC_02317	0.013698368	0.52	UDP-N-acetylmuramoylalanyl-D-glutamyl-2,6-diaminopimelate--D-alanyl-D-alanyl ligase
SAOUHSC_02366	0.045657894	0.5	fructose-bisphosphate aldolase
SAOUHSC_01403	0.049582505	0.5	cold shock protein
SAOUHSC_00086	0.038891504	-0.51	acetoin reductase
SAOUHSC_00779	0.032030545	-0.52	excinuclease ABC subunit B
SAOUHSC_02379	0.027876125	-0.53	2-deoxyribose-5-phosphate aldolase
SAOUHSC_01829	0.009192862	-0.55	30S ribosomal protein S4
SAOUHSC_00524	0.030830051	-0.55	DNA-directed RNA polymerase subunit beta
SAOUHSC_01774	0.036568869	-0.55	porphobilinogen deaminase
SAOUHSC_01749	0.027876125	-0.56	S-adenosylmethionine:tRNA ribosyltransferase-isomerase
SAOUHSC_00100	0.025429839	-0.57	2-deoxyribose-5-phosphate aldolase
SAOUHSC_00642	0.026274147	-0.57	teichoic acid biosynthesis protein
SAOUHSC_02511	0.035608176	-0.57	50S ribosomal protein L4
SAOUHSC_01040	0.041457451	-0.57	pyruvate dehydrogenase complex, E1 component subunit alpha
SAOUHSC_01688	0.047874155	-0.58	GTP-binding protein LepA
SAOUHSC_02512a	0.035377483	-0.59	30S ribosomal protein S10
SAOUHSC_00493	0.046181270	-0.59	lysyl-tRNA synthetase
SAOUHSC_02337	0.016125623	-0.62	UDP-N-acetylglucosamine 1-carboxyvinyltransferase
SAOUHSC_01472	0.008391946	-0.63	DnaQ family exonuclease/DinG family helicase
SAOUHSC_02385	0.025096117	-0.63	mannose-6-phosphate isomerase
SAOUHSC_01146	0.027441749	-0.63	phospho-N-acetylmuramoyl-pentapeptide-transferase
SAOUHSC_00445	0.027246968	-0.65	recombination protein RecR
SAOUHSC_00246	0.038802271	-0.66	drug transporter
SAOUHSC_00719	0.044038731	-0.66	7-cyano-7-deazaguanosine (preQ0) biosynthesis protein QueE
SAOUHSC_01248	0.018893698	-0.67	tRNA pseudouridine synthase B
SAOUHSC_00365	0.009193778	-0.69	alkyl hydroperoxide reductase subunit C
SAOUHSC_00472	0.021087536	-0.69	ribose-phosphate pyrophosphokinase
SAOUHSC_01799	0.011768464	-0.71	histidine kinase
SAOUHSC_01169	0.035377483	-0.71	carbamoyl phosphate synthase small subunit
SAOUHSC_00467	0.001843707	-0.72	pur operon repressor
SAOUHSC_00469	0.015609503	-0.73	regulatory protein SpoVG

SAOUHSC_01757	0.004528955	-0.74	50S ribosomal protein L21
SAOUHSC_00888	0.010981845	-0.74	monovalent cation/H+ antiporter subunit B
SAOUHSC_00802	0.000763933	-0.75	carboxylesterase
SAOUHSC_01247	0.014672841	-0.76	ribosome-binding factor A
SAOUHSC_01043	0.000945008	-0.77	dihydrolipoamide dehydrogenase
SAOUHSC_00454	0.033865137	-0.79	DNA polymerase III subunit delta'
SAOUHSC_02622	0.037437918	-0.8	sodium/glutamate symporter
SAOUHSC_00889	0.003456144	-0.81	monovalent cation/H+ antiporter subunit A
SAOUHSC_02763	0.024629119	-0.82	peptide ABC transporter ATP-binding protein
SAOUHSC_00474	0.005567462	-0.83	50S ribosomal protein L25/general stress protein Ctc
SAOUHSC_00183	0.013491149	-0.84	sugar phosphate antiporter
SAOUHSC_01840	0.009924681	-0.85	transglycosylase domain-containing protein
SAOUHSC_02987	0.029349202	-0.85	accessory Sec system protein Asp2
SAOUHSC_02111	0.003791926	-0.86	DNA polymerase IV
SAOUHSC_01031	0.009717154	-0.88	cytochrome d ubiquinol oxidase subunit I
SAOUHSC_00578	0.000102403	-0.89	mevalonate diphosphate decarboxylase
SAOUHSC_00070	0.000723530	-0.89	accessory regulator-like protein
SAOUHSC_02937	0.001625829	-0.89	choline transporter
SAOUHSC_00804	0.000349820	-0.9	SsrA-binding protein
SAOUHSC_00776	0.000409505	-0.9	excinuclease ABC subunit B
SAOUHSC_00364	0.001095282	-0.9	alkyl hydroperoxide reductase subunit F
SAOUHSC_00652	0.008986402	-0.9	iron compound ABC transporter ATP-binding protein
SAOUHSC_02003	0.001218025	-0.91	ABC transporter ATP-binding/permease
SAOUHSC_02988	0.030013685	-0.91	accessory Sec system protein Asp1
SAOUHSC_03052	0.000563326	-0.92	tRNA uridine 5-carboxymethylaminomethyl modification protein GidA
SAOUHSC_00004	0.000450815	-0.93	recombination protein F
SAOUHSC_00730	0.000914927	-0.94	ATP-dependent DNA helicase RecQ
SAOUHSC_00072	0.002255136	-0.95	lipoprotein SirB
SAOUHSC_01759	0.023458675	-0.95	rod shape-determining protein MreC
SAOUHSC_00887	0.000233888	-0.96	monovalent cation/H+ antiporter subunit C
SAOUHSC_02703	0.000534354	-0.96	2,3-bisphosphoglycerate-dependent phosphoglycerate mutase

SAOUHSC_00654	0.001653663	-0.96	ferrichrome ABC transporter permease
SAOUHSC_00001	0.002156798	-0.96	chromosomal replication initiation protein
SAOUHSC_03013	0.008905013	-0.98	histidinol dehydrogenase
SAOUHSC_02806	0.006314112	-0.99	gluconate permease
SAOUHSC_00366	0.003559610	-1	NAD(P)H-flavin oxidoreductase
SAOUHSC_02430	0.000292331	-1.01	ABC transporter substrate-binding protein
SAOUHSC_00174	0.005526084	-1.01	M23/M37 peptidase domain-containing protein
SAOUHSC_02766	0.020745984	-1.01	peptide ABC transporter permease
SAOUHSC_03015	0.023202746	-1.04	ATP phosphoribosyltransferase regulatory subunit
SAOUHSC_00579	9.53E-07	-1.06	phosphomevalonate kinase
SAOUHSC_01038	0.009127978	-1.06	peptide deformylase
SAOUHSC_00720	0.008577146	-1.07	6-pyruvoyl tetrahydropterin synthase
SAOUHSC_01808	0.000508355	-1.08	acetyl-CoA carboxylase carboxyltransferase subunit alpha
SAOUHSC_01048	0.001920817	-1.08	spermidine/putrescine ABC transporter permease
SAOUHSC_02664	0.001275780	-1.09	transcriptional regulator
SAOUHSC_01809	0.001376786	-1.11	acetyl-CoA carboxylase carboxyltransferase subunit beta
SAOUHSC_00071	0.002171498	-1.13	lipoprotein SirC
SAOUHSC_02300	0.038744118	-1.13	STAS domain-containing protein
SAOUHSC_00384	0.032007543	-1.14	superantigen-like protein
SAOUHSC_02933	0.000171390	-1.15	betaine aldehyde dehydrogenase
SAOUHSC_00721	0.004342558	-1.15	7-cyano-7-deazaguanine synthase QueC
SAOUHSC_02874	0.000352557	-1.16	cation transporter E1-E2 family ATPase
SAOUHSC_02808	0.000427276	-1.16	gluconate kinase
SAOUHSC_02737	0.000052377	-1.17	epimerase/dehydratase
SAOUHSC_01582	0.000009284	-1.18	bacteriophage integrase
SAOUHSC_02542	0.003389420	-1.18	molybdopterin biosynthesis protein MoeA
SAOUHSC_02299	0.007931505	-1.18	serine-protein kinase RsbW
SAOUHSC_02932	0.000011119	-1.19	choline dehydrogenase
SAOUHSC_02682	0.000855625	-1.19	uroporphyrin-III C-methyltransferase
SAOUHSC_00005	4.53E-06	-1.2	DNA gyrase subunit B
SAOUHSC_00008	0.010426250	-1.2	histidine ammonia-lyase
SAOUHSC_00006	1.40E-07	-1.21	DNA gyrase subunit A
SAOUHSC_02365	0.000210831	-1.21	UDP-N-acetylglucosamine 1-carboxyvinyltransferase
SAOUHSC_02754	0.000543097	-1.21	ABC transporter ATP-binding protein

SAOUHSC_00236	0.003419639	-1.21	6-phospho-beta-glucosidase
SAOUHSC_00625	0.007400756	-1.21	monovalent cation/H+ antiporter subunit A
SAOUHSC_02764	0.004687757	-1.22	peptide ABC transporter ATP-binding protein
SAOUHSC_01705	0.026142612	-1.22	enterotoxin family protein
SAOUHSC_02098	0.034620536	-1.22	DNA-binding response regulator VraR
SAOUHSC_02312	0.002026073	-1.23	potassium-transporting ATPase subunit A
SAOUHSC_00849	0.000024047	-1.24	aminotransferase
SAOUHSC_00998	0.000695424	-1.24	methicillin resistance protein FmtA
SAOUHSC_02550	0.000582613	-1.25	formate dehydrogenase accessory protein
SAOUHSC_00491	0.001378570	-1.26	2-amino-4-hydroxy-6-hydroxymethyldihydropteridine pyrophosphokinase
SAOUHSC_02099	0.020211008	-1.26	histidine kinase
SAOUHSC_02154	5.53E-07	-1.27	ABC transporter ATP-binding protein
SAOUHSC_02389	0.000507743	-1.28	cation efflux family protein
SAOUHSC_02565	0.013168866	-1.28	urease accessory protein UreD
SAOUHSC_00577	1.48E-06	-1.29	mevalonate kinase
SAOUHSC_00175	0.000338660	-1.31	multiple sugar-binding transport ATP-binding protein
SAOUHSC_02765	0.005682835	-1.31	nickel ABC transporter permease
SAOUHSC_02678	4.21E-09	-1.32	respiratory nitrate reductase subunit gamma
SAOUHSC_00434	0.003278541	-1.32	LysR family transcriptional regulator
SAOUHSC_00291	0.011097221	-1.32	PfkB family carbohydrate kinase
SAOUHSC_00636	0.000601947	-1.33	iron (chelated) ABC transporter permease
SAOUHSC_01032	0.000698742	-1.33	cytochrome d ubiquinol oxidase subunit II
SAOUHSC_03009	0.001101758	-1.34	1-(5-phosphoribosyl)-5-[(5-phosphoribosylamino)methylidene amino] imidazole-4-carboxamide isomerase
SAOUHSC_03051	5.57E-07	-1.36	16S rRNA methyltransferase GidB
SAOUHSC_00653	0.000591014	-1.36	ferrichrome transport permease FhuB
SAOUHSC_00729	1.55E-09	-1.37	ABC transporter ATP-binding protein
SAOUHSC_02152	3.40E-09	-1.39	ABC transporter ATP-binding protein
SAOUHSC_01552	0.020697699	-1.39	bacteriophage L54a deoxyuridine 5-triphosphate nucleotidohydrolase
SAOUHSC_00847	0.001284743	-1.4	ABC transporter ATP-binding protein

SAOUHSC_00628	0.004118526	-1.41	monovalent cation/H+ antiporter subunit D
SAOUHSC_00466	3.29E-06	-1.44	4-diphosphocytidyl-2C-methyl-D-erythritol kinase
SAOUHSC_02708	0.002952575	-1.44	gamma-hemolysin h-gamma-II subunit
SAOUHSC_02803	0.001279344	-1.45	fibronectin-binding protein
SAOUHSC_03055	0.000036568	-1.46	50S ribosomal protein L34
SAOUHSC_03053	0.000128723	-1.46	tRNA modification GTPase TrmE
SAOUHSC_00340	0.000011623	-1.48	trans-sulfuration enzyme family protein
SAOUHSC_01165	0.000049752	-1.51	uracil permease
SAOUHSC_03054	0.000605063	-1.52	ribonuclease P
SAOUHSC_02453	2.27E-10	-1.55	tagatose-6-phosphate kinase
SAOUHSC_00097	5.99E-06	-1.55	purine nucleoside phosphorylase
SAOUHSC_01686	0.000013669	-1.56	coproporphyrinogen III oxidase
SAOUHSC_02455	5.635E-10	-1.61	galactose-6-phosphate isomerase subunit LacA
SAOUHSC_02839	1.648E-09	-1.61	L-serine dehydratase, iron-sulfur-dependent subunit alpha
SAOUHSC_00339	2.53E-07	-1.62	bifunctional homocysteine S-methyltransferase/5,10-methylenetetrahydrofolate reductase
SAOUHSC_00338	1.519E-06	-1.63	5-methyltetrahydropteroyltriglutamate--homocysteine S-methyltransferase
SAOUHSC_T00029	8.43E-06	-1.63	tRNA-Ile
SAOUHSC_02454	1.48E-12	-1.64	galactose-6-phosphate isomerase subunit LacB
SAOUHSC_00178	2.41E-07	-1.64	maltose ABC transporter permease
SAOUHSC_02680	4.77E-06	-1.64	nitrate reductase subunit beta
SAOUHSC_00794	0.002202797	-1.64	glycolytic operon regulator
SAOUHSC_02679	3.83E-07	-1.65	respiratory nitrate reductase subunit delta
SAOUHSC_00632	0.001186141	-1.65	monovalent cation/H+ antiporter subunit G
SAOUHSC_02451	7.99E-11	-1.66	PTS system lactose-specific transporter subunit IIA
SAOUHSC_T0001	0.002171498	-1.66	tRNA-Ala
SAOUHSC_02452	2.27E-10	-1.7	tagatose 1,6-diphosphate aldolase
SAOUHSC_00556	1.26E-07	-1.7	proline/betaine transporter
SAOUHSC_02681	0.000028682	-1.7	nitrate reductase subunit alpha
SAOUHSC_T0006	4.62E-06	-1.71	tRNA-Arg
SAOUHSC_02080	3.00E-11	-1.72	bacteriophage L54a antirepressor
SAOUHSC_00177	1.32E-08	-1.72	maltose ABC transporter permease
SAOUHSC_02078	6.74E-09	-1.76	phi PV83 orf 10-like protein

SAOUHSC_01601	1.14E-18	-1.86	alpha-D-1,4-glucosidase
SAOUHSC_02544	0.000139232	-1.87	molybdopterin precursor biosynthesis MoaB
SAOUHSC_00176	4.73E-14	-1.9	extracellular solute-binding protein
SAOUHSC_02562	0.000706412	-1.91	urease accessory protein UreE
SAOUHSC_02450	3.767E-12	-1.92	PTS system lactose-specific transporter subunit IIBC
SAOUHSC_01691	0.000659598	-1.92	DNA internalization-related competence protein ComEC/Rec2
SAOUHSC_02989	0.004200843	-1.93	accessory Sec system protein translocase subunit SecY2
SAOUHSC_00773	1.44E-06	-1.94	LysM domain-containing protein
SAOUHSC_02330	0.000060807	-1.97	phosphomethylpyrimidine kinase
SAOUHSC_01602	2.15E-13	-1.98	maltose operon transcriptional repressor
SAOUHSC_01690	0.000011826	-2.01	DNA polymerase III subunit delta
SAOUHSC_03033	1.63E-12	-2.03	high affinity nickel transporter
SAOUHSC_T00025	2.32E-12	-2.09	tRNA-Gly
SAOUHSC_02047	0.000087378	-2.11	phage head morphogenesis protein
SAOUHSC_R0001	0.001653805	-2.11	16S Ribosomal RNA
SAOUHSC_00629	0.003453695	-2.11	monovalent cation/H+ antiporter subunit E
SAOUHSC_02061	2.39E-07	-2.13	phi PVL orf 50-like protein
SAOUHSC_02449	8.924E-12	-2.14	6-phospho-beta-galactosidase
SAOUHSC_00359	0.000168680	-2.15	phosphoglycerate mutase family protein
SAOUHSC_02564	0.000016339	-2.22	urease accessory protein UreG
SAOUHSC_01761a	4.44E-06	-2.23	membrane protein
SAOUHSC_02053	0.032170316	-2.27	transcriptional activator rinb-like protein
SAOUHSC_R0008	0.048290429	-2.31	23S Ribosomal RNA
SAOUHSC_02873	1.74E-10	-2.35	cation transporter E1-E2 family ATPase
SAOUHSC_00626	0.000322471	-2.35	monovalent cation/H+ antiporter subunit B
SAOUHSC_02216	4.08E-17	-2.44	phage DnaC-like protein
SAOUHSC_02219	2.46E-12	-2.46	phi ETA orf 20-like protein
SAOUHSC_T00051	0.000780041	-2.48	tRNA-Ser
SAOUHSC_R00016	0.000860502	-2.49	5S Ribosomal RNA
SAOUHSC_02217	2.75E-15	-2.52	phi ETA orf 22-like protein
SAOUHSC_02563	1.52E-06	-2.57	urease accessory protein UreF
SAOUHSC_02048	4.20E-06	-2.6	SPP1 family phage portal protein
SAOUHSC_02609	0.007686790	-2.87	fosfomycin resistance protein FosB
SAOUHSC_00412	2.26E-09	-2.88	NADH dehydrogenase subunit 5

SAOUHSC_T0004 1	0.015035536	-2.9	tRNA-Met
SAOUHSC_03005	0.000672437	-2.94	intercellular adhesion protein C
SAOUHSC_02864	7.04E-23	-3.01	ferrous iron transport protein B
SAOUHSC_02213	0.000019947	-3.04	phi ETA orf 25-like protein
SAOUHSC_02561	8.69E-12	-3.05	urease subunit alpha
SAOUHSC_02559	6.02E-09	-3.21	urease subunit beta
SAOUHSC_T0001 4	1.10E-09	-3.3	tRNA-Asp
SAOUHSC_02051	1.25E-18	-3.31	int gene activator RinA
SAOUHSC_T0005 3	0.034087313	-3.47	tRNA-Thr
SAOUHSC_02064	0.000815298	-3.53	phi ETA orf 25-like protein
SAOUHSC_02558	1.873E-15	-3.91	urease subunit gamma
SAOUHSC_R0004	5.52E-08	-3.96	16S Ribosomal RNA
SAOUHSC_T0003 5	0.001709687	-4.19	tRNA-Lys
SAOUHSC_T0005 8	0.023909467	-4.33	tRNA-Tyr
SAOUHSC_R0005	0.049173730	-4.39	16S Ribosomal RNA
SAOUHSC_T0004 6	0.023640117	-4.65	tRNA-OTHER
SAOUHSC_T0002 1	0.005234052	-7.14	tRNA-Gly

Table S5. Details of PCR primers used for qPCR.

Primer name	Sequence（5′to 3′）
01516-F	agtccgataccagtagatgaaga
01516-R	gcttgccctgttgcttttct
01529-F	gaagccccagaacctacaaaa
01529-R	ctgaatgatcttcactgtccgt
02064-F	tggaaggaagggaaactggt
02064-R	ccatccattcttgacctgtgt
00188-F	tcggctggatgtgctaatga
00188-R	gcgcgaagttaaggatgtgt

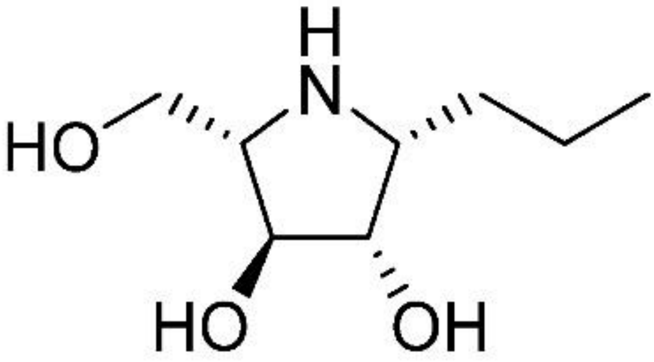

Figure S1. The chemical structure of PDIA.

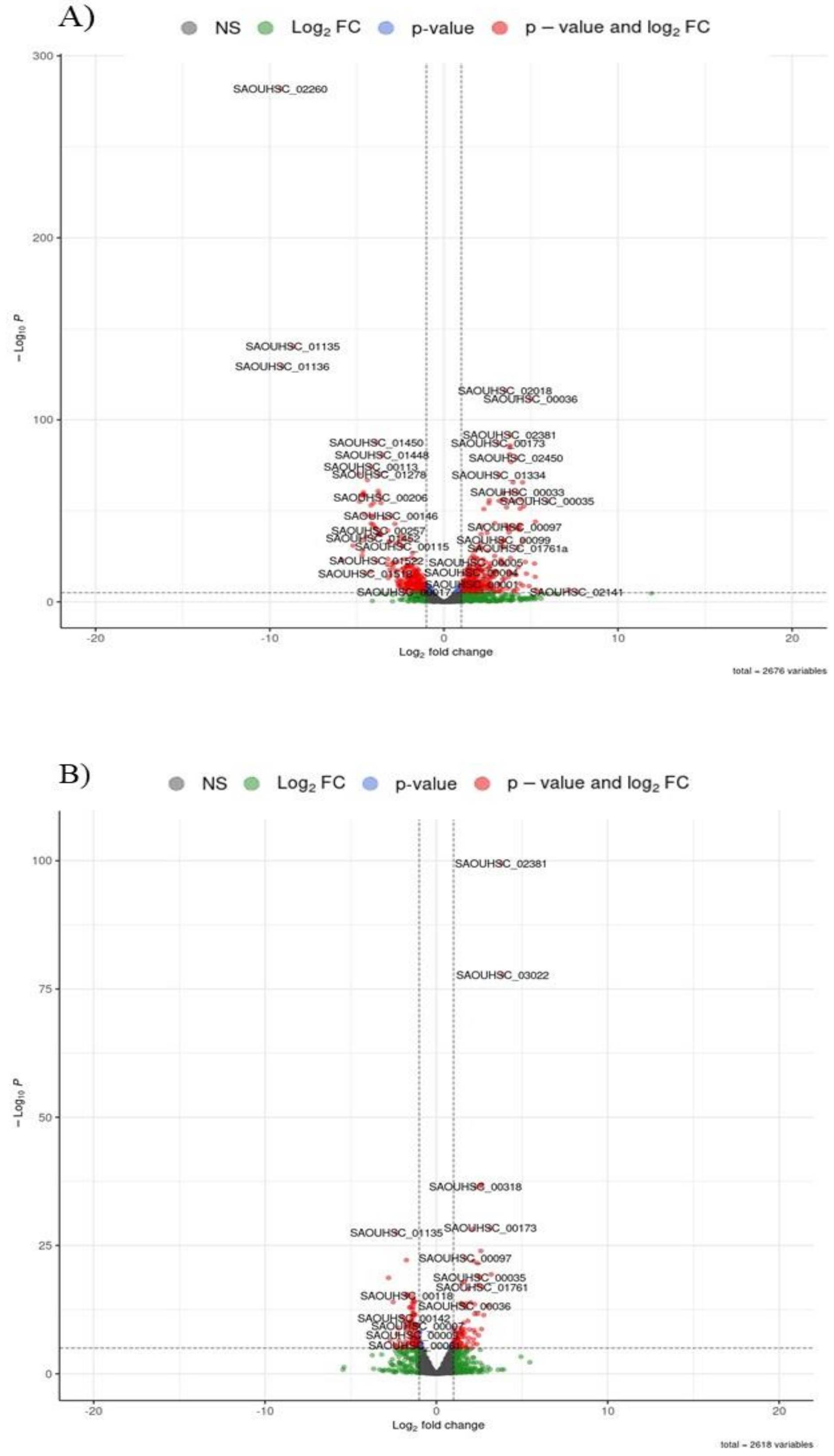

Figure S2. Volcano graph generated for detected differentially expressed genes in early biofilm (A) and mature biofilm (B) of *S. aureus*, comparing the control culture with the culture in the presence of PDIA iminosugar.

Figure S3. Functional comparison of DEGs in the PDIA treated-group compared to control group in early- and mature biofilm. Functions are organized into three groups, namely Biological processes, Cellular components, and Molecular functions

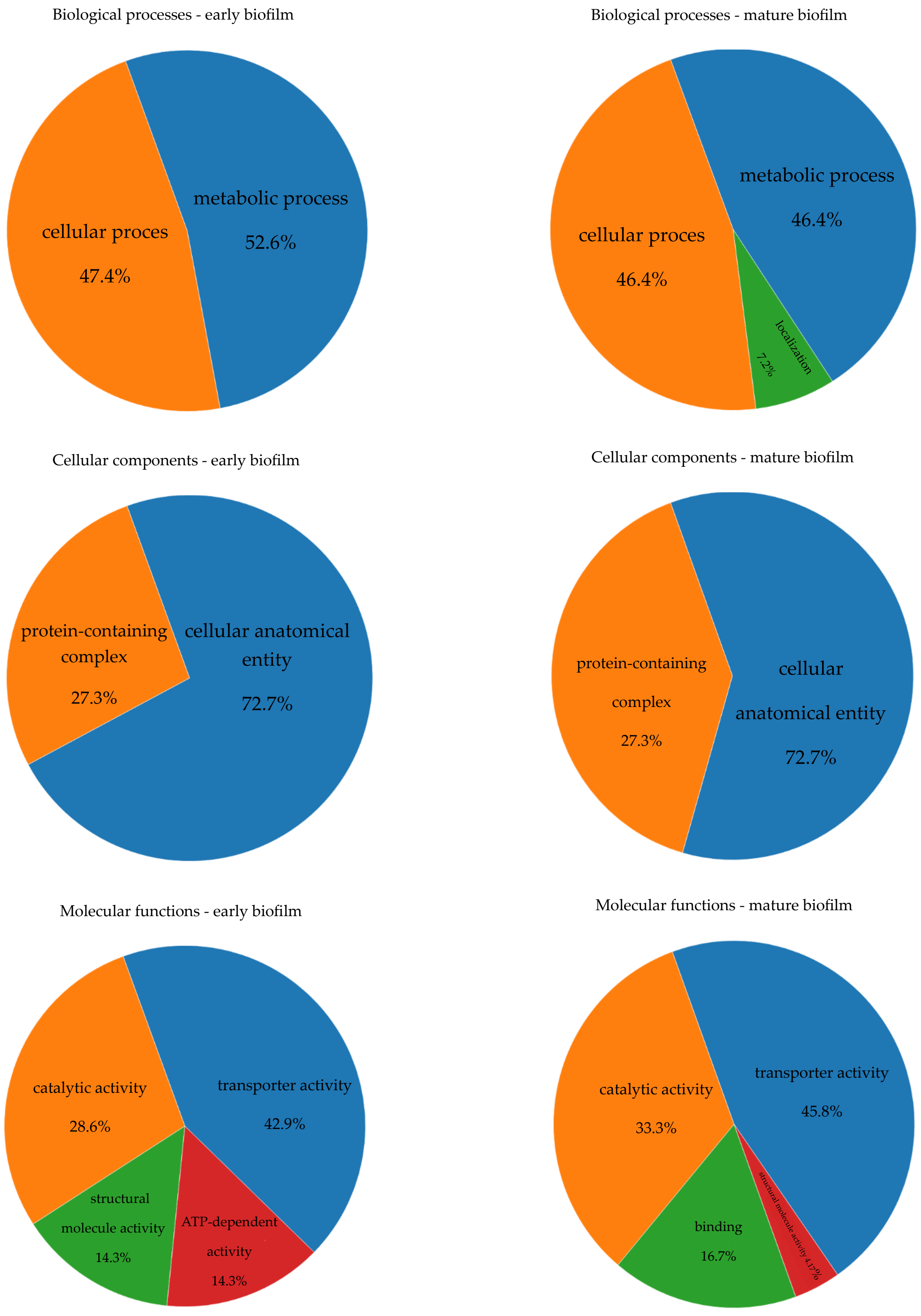

Supplement: Supplementary file 1 [file antibiotics-14-00668-s001.zip › antibiotics-3620706-supplementary.pdf]
